# Biofilm‐Controlled Molecular Crosstalk: Nitrogen Fixation, Arsenic Transformation and Antibiotic Resistance

**DOI:** 10.1155/ijm/4307970

**Published:** 2026-11-04

**Authors:** Dhritidipa Bagchi, Arnab Majumdar, Ioly Kotta-Loizou, Martin Buck

**Affiliations:** ^1^ Department of Microbiology West Bengal State University Kolkata India wbsubregistration.org; ^2^ Department of Life Sciences Imperial College London London UK imperial.ac.uk

**Keywords:** antibiotic resistance, arsenic induction, diazotrophy, EPS, gene transfer

## Abstract

Biological nitrogen fixation, arsenic biotransformation and antibiotic resistance are conventionally studied separately, yet the environmental biofilm—bacteria′s dominant organisational state in soils, sediments and rhizospheres—supplies a shared physicochemical and genetic context in which the three can intersect. The extracellular polymeric substance (EPS) matrix imposes steep oxygen, redox and metabolite gradients across micrometre scales and demonstrably shelters oxygen‐sensitive nitrogenase, retains arsenic‐transforming enzymes (ArsC, ArsM and AioA) and elevates horizontal gene transfer (HGT) frequencies orders of magnitude above planktonic conditions. Individual circuits are genetically established in single organisms—RpoN coactivates pslA and nifA and the non‐coding RNA NfiS stabilises nifK and katB transcripts, in *Pseudomonas stutzeri* A1501—but their assembly into one programme linking surface commitment, diazotrophic activation and oxidative stress defence, with additional c‐di‐GMP and ArsR stimulon input under arsenic stress, is proposed, not demonstrated. Arsenic‐driven coselection of antibiotic resistance genes (ARGs) is the best‐supported limb, operating through cross‐resistance (RND efflux), co‐resistance (ars–ARG colocation on Tn21, Class 1 integrons, IncP/IncQ plasmids) and coregulation (ArsR stimulon, SOS‐derepressed intI1), whereas the proposal that diazotrophy‐derived ammonium supports S‐adenosyl‐l‐methionine supply for ArsM‐mediated methylation rests on gene coenrichment and rate correlation rather than direct flux measurement. This review integrates these processes into a single biofilm framework, classifies each proposed linkage by the evidence behind it, identifies candidate trifunctional mobile elements carrying ars, nif and ARG cassettes in hyperaccumulator rhizospheres and paddy soils and specifies the minimum spatially resolved single‐cell dataset (bacterial‐MERFISH, par‐seqFISH and SIP‐NanoSIMS) needed to test the proposed couplings.

## 1. Introduction

Biological nitrogen fixation (BNF) is the primary natural pathway by which atmospheric N_2_ enters the bioavailable nitrogen pool, contributing approximately 0.9–1.3 × 10^14^ g N per year to global soils [1]. The nitrogenase complex responsible is among the most energetically costly enzymatic reactions in biology—requiring 16 ATP per N_2_ reduced—and is irreversibly inactivated by molecular oxygen, fundamentally constraining diazotrophy to low‐oxygen microenvironments with adequate carbon supply [2]. Arsenic (As) contamination of groundwater and soil constitutes a global public health emergency of equivalent scale. An estimated 94–220 million people are currently exposed to As levels exceeding the WHO guideline of 10 *μ*g/L, with the greatest burden concentrated in South, Southeast and Central Asia and parts of South America [3]. As enters ecosystems through both geogenic mobilisations from aquifer sediments and anthropogenic activities including mining, smelting and arsenical pesticide use. Its environmental fate is governed by microbially mediated interconversions between As(V) [As(V)] and arsenite [As(III)], methylation to dimethylarsinic acid (DMA) and monomethylarsonic acid (MMA) and volatilisation to gaseous arsines—reactions that collectively control As speciation, mobility and ecotoxicology across environmental compartments [3, 4]. The third dimension of the triad is antibiotic resistance (AR). Antibiotic resistance genes (ARGs) are pervasive environmental contaminants disseminated via horizontal gene transfer (HGT) on mobile genetic elements (MGEs) including plasmids, integrative and conjugative elements, transposons and integrons [5]. Critically, As exerts coselective pressure on microbial communities, favouring ARG persistence through cross‐resistance (shared efflux pumps or oxidative stress responses), coresistance (ARGs and As resistance genes colocated on shared MGEs) and coregulation (shared transcriptional regulators responding to both stressors) [5, 6]. As exposure additionally enhances biofilm formation in exposed strains, a phenotype that independently amplifies antimicrobial resistance [7, 8].

The convergence of BNF, As transformation and AR is most consequential in specific biogeochemical niches. In the rhizosphere of the As hyperaccumulator *Pteris vittata*, As stress paradoxically enhanced nitrogen cycling and diazotrophic recruitment, with rhizospheric N indices (total N, NH_4_^+^, NO_3_^−^) positively correlated with As concentrations [9]. In the rhizosphere of *Vetiveria zizanioides*, As‐methylating organisms bearing arsM are specifically coenriched with organisms carrying nitrogen fixation genes (nifD and nifK), with As methylation rates positively correlated with N_2_ fixation rates [10]. The mechanistic basis appears to reside in nitrogen fixation‐derived ammonium stimulating S‐adenosyl‐l‐methionine (SAM) synthesis—the universal methyl donor for ArsM‐type methyltransferases—creating a functional metabolic coupling between diazotrophy and As detoxification [11]. In paddy soils, seasonal flooding creates strongly reducing conditions that simultaneously mobilise As(III) and generate microoxic–anoxic zones favouring both diazotrophy and anaerobic As‐transforming metabolisms [12]. Under combined As and antibiotic pressure, As stress significantly elevated ARG abundance and MGE prevalence (including intI1 integrase), whereas As biotransformation genes (ABGs) (aioA and arsM) responded to antibiotic exposure concentrations—demonstrating interactive rather than independent selective dynamics [6, 13]. In maize rhizosphere experiments, antibiotic additions altered nitrate and nitrite reductase activities, modified NH_4_^+^ and NO_3_^−^ concentrations and shifted As(V)/As(III) distributions in both rhizosphere soil and root tissue, illustrating the tridirectional nature of the triad in a single ecological context [7, 8]. In groundwater systems, As cycling is tightly coupled to iron, sulphur and nitrogen redox transformations [14], and biofilm‐based engineered systems—constructed wetlands, fixed‐bed reactors, IFAS systems—demonstrate the tractability of coupling these biogeochemical processes for simultaneous nitrate and As removal [15–17]. The co‐occurrence of ARGs, ars operons and nif gene clusters on shared MGEs within environmental biofilms raises a critical concern: As contamination may stabilise and propagate the resistome within diazotrophic communities, with cascading consequences for soil nitrogen fertility and food security [6, 13].

Most mechanistic understanding of this triad has been developed from planktonic cultures, which fundamentally misrepresent the dominant organisational state of bacteria in environmentally relevant systems. In soils, sediments, rhizospheres, aquifer sediments and engineered treatment systems, microbial biomass overwhelmingly exists as surface‐attached communities embedded within extracellular polymeric substance (EPS) matrices—namely, biofilms [18]. Biofilm architecture generates steep gradients of oxygen, nutrients, pH and metabolites across micrometre scales, creating physiologically stratified subpopulations that simultaneously sustain aerobic respiration, microaerobic nitrogen fixation and anaerobic As‐transforming metabolism—activities mutually exclusive in homogeneous planktonic cultures [19, 20]. Anoxic interior zones protect nitrogenase from oxidative inactivation [2, 21], whereas dense cell proximity and the eDNA‐rich EPS matrix elevate HGT frequencies by orders of magnitude, accelerating codissemination of ARGs and ars genes [22]. Crucially, the triad is molecularly interconnected through shared signalling circuits activated by surface commitment itself. The second messenger cyclic di‐GMP (c‐di‐GMP)—master regulator of the motile‐to‐sessile transition—intersects with nitrogen regulatory circuits in diazotrophic pseudomonads and rhizobia [23]. As stress modulates c‐di‐GMP‐mediated biofilm formation in As(III)‐oxidising bacteria [7, 8], and antibiotic‐induced SOS responses promote ARG transfer while influencing EPS production and biofilm persistence. These overlapping regulatory axes indicate that the triad responds coherently to environmental stress, rendering the biofilm both the most relevant ecological context and the most appropriate molecular unit of analysis.

This article is a narrative, mechanism‐focused review rather than a systematic review, and the literature base was assembled accordingly. Web of Science Core Collection, Scopus, PubMed and Google Scholar were searched for records published between January 2000 and February 2026, supplemented by hand‐searching the reference lists of retrieved reviews and by forward citation tracking of key primary papers [24, 25]. Foundational biochemical and genetic studies published before 2000 were retained where they remain the primary source for a mechanism, as for the early characterisation of nitrogenase catalysis and of the ars operon. Searches combined terms from four blocks with Boolean operators (i) biofilm OR ‘extracellular polymeric substance’ OR EPS OR aggregate OR floc OR ‘microbial mat’; (ii) ‘nitrogen fixation’ OR diazotroph ^∗^ OR nitrogenase OR nifH OR nifA OR RpoN OR ‘sigma‐54’; (iii) arsenic OR arsenite OR arsenate OR arsM OR arsR OR arsC OR aioA OR ‘arsenic methylation’; and (iv) ‘antibiotic resistance’ OR ARG OR ‘horizontal gene transfer’ OR conjugation OR integron OR ‘mobile genetic element’ OR persister. Searches targeting regulatory mechanisms additionally used c‐di‐GMP, ‘quorum sensing’, Gac/Rsm, RsmA, NfiS, ‘ArsR stimulon’, SOS, recA and intI1. Primary experimental studies were prioritised for every mechanistic statement; reviews and meta‐analyses are cited for consensus background, for quantitative syntheses or where a review is itself the primary source of a conceptual framework. Preference was given to studies reporting direct functional measurements—^15^N_2_ or acetylene‐reduction rates, purified‐protein biochemistry, defined mutants with complementation, quantified transfer frequencies and speciation by hyphenated chromatography or synchrotron spectroscopy—over studies reporting marker‐gene abundance alone. Correlative community‐level surveys were retained where they define the environmental context but are identified as correlative in the text. Records not available in English, conference abstracts without accessible primary data and outputs of journals not indexed in the databases above were excluded. Because this search strategy is deliberately targeted rather than exhaustive, statements that a particular measurement has not been reported are given throughout in the form ‘to our knowledge, and within the scope of the search, we did not identify a study that …’ rather than as absolute claims of absence.

This review examines the molecular mechanisms underpinning biofilm‐mediated crosstalk between nitrogen fixation, As transformation and AR, focusing deliberately on shared regulatory circuits, signalling cascades, matrix properties and gene transfer mechanisms. Ecological and bioremediation literature is referenced where it provides mechanistic context, with the review proceeding from biofilm biology through the molecular biology of each triad component to their functional integration and an agenda for resolving critical mechanistic knowledge gaps. Table 1 lists out the major linkage of these combinations that are either experimentally tested or hypothesised.

**Table 1:** Evidence status of each proposed linkage within the nitrogen fixation–arsenic–antibiotic resistance triad.

No.	Proposed linkage	Mechanistic basis invoked	Strongest current evidence (system; measurement)	Class	Decisive test
	Biofilm architecture → diazotrophy				

1	EPS‐generated O_2_ gradients shelter nitrogenase	Diffusion‐limited O_2_ consumption in aggregate cores	Aggregate‐forming diazotrophs; rosette‐like aggregates with nitrogenase concentration 600‐fold higher in hypoxic cores [21]; microoxic cores in alga‐colonising Planctomycetota [26]	A	Already established; extend to soil diazotroph biofilms with O_2_ microsensors plus ^15^N_2_
2	RpoN coactivates *pslA* (EPS) and *nifA* from adjacent *σ* ^54^‐dependent promoters	Shared *σ* factor under N‐limited microoxia	*P. stutzeri* A1501; transcriptional coactivation [27]	A (single strain)	Promoter occupancy (ChIP‐seq) plus *rpoN* complementation in a second diazotroph genus
3	Anr links the biofilm developmental programme to nitrogenase activation	O_2_‐labile [4Fe–4S] FNR‐family sensor	*anr* disruption impaired *nifH*/*nifC* expression (qRT‐PCR) and reduced biofilm formation [28]	A (single strain)	As above; spatially resolved *anr* reporter across biofilm depth
4	NfiS ncRNA couples diazotrophy to oxidative stress defence	Base‐pairing stabilisation of *nifK* and *katB* mRNAs	*P. stutzeri* A1501; stem‐loop mutants ([29]; [30])	A (single strain)	Test *nfiS* function under arsenic‐generated ROS
5	Gac/Rsm–c‐di‐GMP (SadC/BifA) coregulation of Psl and nitrogen fixation	RsmA derepression of *pslA*; c‐di‐GMP as surface commitment switch	*P. stutzeri* A1501 [27]; c‐di‐GMP switch logic from *P. aeruginosa* [23]	A/B	Quantify c‐di‐GMP pools with simultaneous ^15^N_2_ rates in *sadC*/*bifA* mutants
6	AHL quorum sensing controls EPS and symbiotic nitrogen fixation	LuxI/LuxR density sensing; EPS II recognition by host	Rhizobial QS mutants show aberrant infection threads and reduced fixation efficiency [31]	**A** (symbiotic)	—
7	AHL quorum sensing directly regulates *nif* expression in free‐living diazotrophs	QS‐regulated EPS → O_2_ gradient → Anr/NifL feedback	None; the manuscript states this explicitly as an open question	D	*LuxI*‐homologue deletion in *A. brasilense* with *nif* reporter and O_2_ microprofiling
	Arsenic ↔ diazotrophy				

8	Arsenic suppresses nitrogen fixation	*NifH* transcript loss; EPS reduction	Rice‐field *β*‐, *γ*‐Proteobacterial and Firmicute diazotrophs; *nifH* transcripts significantly suppressed with concurrent EPS decrease [32]	A	Protein‐level nitrogenase and ^15^N_2_ rates across an arsenic dose series
9	The suppression acts upstream via shared RpoN/Gac‐Rsm control rather than parallel targets	Coregulation of *nifH* and EPS	Inference from the cosuppression pattern only	D	*rpoN*/*rsmA* backgrounds under arsenic; promoter‐level readout
10	As(III) inactivates nitrogenase by displacing Fe from Fe–S clusters	Thiophilic As(III) attack on [4Fe–4S]	Demonstrated for aconitase and succinate dehydrogenase; nitrogenase Fe protein by extension [33]	**B**	In vitro As(III) titration of purified Fe protein with EPR and activity assay
11	Arsenic actively selects and recruits diazotrophs in hyperaccumulator rhizospheres	Root exudate‐mediated selection	*P. vittata*; N indices correlated with As, homogeneous selection rising 41.6% → 54.9% along the gradient with a 42‐ASV core module [9, 34]	**C**	Reciprocal transplant or exudate‐amendment microcosm with rate measurements
12	Diazotrophy‐derived ammonium sustains SAM supply for ArsM methylation	NH_4_^+^ → glutamine → methionine → SAM	*V. zizanioides* rhizosphere; *arsM* coenriched with *nifD*/*nifK*, methylation and fixation rates correlated in root compartments but not bulk soil [10]	**C**	^15^NH_4_^+^/^13^C‐methionine tracing to SAM and methylated As pools; *nif* mutant with ammonium rescue
	Arsenic ↔ antibiotic resistance				

13	*ars*–ARG coresistance on shared mobile elements	Physical colocation on Tn*21*, Class 1 integrons, IncP/IncQ/IncW plasmids	Plasmid‐resolved and functional metagenomics of As‐contaminated rhizospheres; IncP‐1*α* carrying *arsRBC* on Tn*402* with *aadB* and *bla* ([35]; [36])	A (genetic colocation)	Conjugation assays showing cotransfer of *ars* and ARG cassettes with matched controls
14	Field‐scale arsenic/metal exposure raises ARG abundance through MGE proliferation	Coselection	50 urban soils, path analysis with MGEs as mediator [37]; poultry‐farm soils [38]; As‐contaminated paddy soils, qPCR [6]	**C**	MIC shifts or *ars* + ARG plasmid enrichment against matched low‐As controls,
15	Coselection persists after antibiotic withdrawal	Nondegrading selector; plasmid/integron maintenance	Source‐water arsenic pollution accident; resistance stability tracked after the event [39]	C	Long‐term mesocosm with antibiotic cessation and As gradient
16	Cross‐resistance through shared RND efflux and oxidative stress responses	Promiscuous efflux substrate range	Demonstrated in nondiazotrophic, largely planktonic systems	B	Efflux‐mutant panel challenged with As and antibiotics in a diazotroph
17	Arsenic‐induced ROS → SOS → *intI1* derepression	RecA‐dependent integrase induction	SOS‐dependent transfer characterised in *E. coli*, where SOS activation and conjugation were mechanistically separable [40]	**B**	As exposure in *recA*‐null diazotroph background with integrase and transfer readouts
	Biofilm → gene mobility				

18	Biofilms amplify horizontal gene transfer	EPS confinement, T4SS contact time, eDNA competence	Conjugative transfer 700‐fold above liquid culture [41]; RP4 retransfer cascades with > 46% permissive genera	A (nondiazotrophic biofilms)	Repeat in a diazotrophic rhizosphere biofilm
19	Arsenic remodels EPS towards eDNA‐ and uronic‐acid‐rich matrices, raising transfer	Matrix composition control of conjugation	As‐driven EPS compositional change measured [42]; EPS depletion reduced conjugation independently of ROS [43]	B	*pslΔ*/*algDΔ*/*pelBΔ* mutants under an As gradient with transfer frequency as readout
20	eDNA from lysed diazotrophs at the oxic–anoxic interface enables joint acquisition of *nif*, *ars* and ARGs	Spatial coincidence of lysis, competence and cassette availability	None; spatial argument only	D	Depth‐resolved eDNA sequencing plus natural‐transformation assays at the interface
21	Trifunctional mobile elements carrying *ars*, *nif* and ARGs	Modular plasmid/ICE cargo assembly	*ars* + ARG + PGP(*nif*) clusters assembled from long‐read plasmid contigs; the manuscript states *nif*‐carrying variants await long‐read confirmation [35]	C/D	Closed long‐read plasmid/ICE genomes demonstrating all three cassette classes on one replicon
	Nitrogen fixation ↔ antibiotic resistance				

22	Diazotrophs are themselves ARG carriers, so selection on either trait acts on the same genomes	Genomic linkage within single lineages		A/C	—
23	Shared regulators (RpoN, NtrBC, (p)ppGpp and Anr) couple nitrogen status to tolerance and efflux	Regulatory pleiotropy	Mechanisms established separately in diazotrophic (*nif* arm) and nondiazotrophic (tolerance arm) systems	B	Tolerance assays in *rpoN*, *relA*/*spoT* and *anr* mutants of a diazotroph under N‐limited microoxia
24	Nitrogenase‐active and antibiotic‐tolerant subpopulations occupy the same anoxic biofilm zone	Shared low‐O_2_, low‐energy niche	Spatial argument; the manuscript notes co‐occurrence should not be read as a pairwise relationship	D	Bacterial‐MERFISH or par‐seqFISH coimaging of *nif* expression and tolerance markers in one biofilm
25	Nitrogen fixation and resistance compete at the single‐cell level but can be reconciled by division of labour	ATP, SAM and reductant competition	Heterocyst impairment in phage‐resistant cyanobacteria [44]; genome‐scale incompatibility signal across > 15,000 genomes [45]	B/C	two‐, four‐, eight‐ and 16‐member consortia with joint nitrogenase and ARG load measured
Class	Definition				

A	Direct experimental evidence obtained in a diazotrophic and/or arsenic‐exposed system—genetic (mutant/complementation), transcriptomic with phenotype or biochemical				
B	Mechanism demonstrated rigorously, but in a nondiazotrophic model organism, a planktonic culture or without arsenic; transferred here by analogy				
C	Field or community‐scale co‐occurrence, correlation or enrichment only; causality not established				
D	Mechanistic inference from established chemistry or regulatory logic; not yet tested in any system				

## 2. Biofilm as a Molecular Reactor

### 2.1. The Biofilm Cycle and Motile‐to‐Sessile Transition Under Spatial Heterogeneity

The planktonic‐to‐biofilm transition involves wholesale reprogramming of gene expression, motility cessation and EPS biosynthesis initiation, progressing through five canonical stages (Figure 1): reversible attachment, irreversible attachment, microcolony formation, mature biofilm development and regulated dispersal triggered by nutrient replenishment or nitric oxide [18]. Critically, the biofilm phenotype is defined by EPS encasement and physiological stratification—not surface attachment—as free‐floating aggregates and microbial flocs recapitulate the same essential properties, enabling aggregate‐forming diazotrophs to generate low‐oxygen microenvironments (Figure 1C) for nitrogenase activity without physical surface contact [2, 21]. The master switch governing this transition is c‐di‐GMP, which at high concentrations represses flagellar motility via YcgR sequestration, induces EPS biosynthesis operons and activates biofilm‐specific transcription factors including VpsR/VpsT and WspR—with the c‐di‐GMP‐mediated surface commitment pathway strongly conserved across diazotrophic lineages including rhizobia and *Pseudomonas* [23]. Surface commitment coinduces antimicrobial tolerance in *P. aeruginosa*, the SagS–BfiSR–BfmRS–MifRS phosphorelay upregulates *β*‐lactamase (blaP1), the multidrug resistance regulator BrlR and efflux pump components within hours of surface contact [18]. In *Pseudomonas stutzeri* A1501, the oxygen‐sensing Anr transcription factor—an FNR‐family regulator with an oxygen‐labile [4Fe–4S] cluster—simultaneously activates nif gene expression and biofilm‐associated regulatory programmes under microoxic conditions, constituting a regulatory coactivation of diazotrophic and sessile phenotypes driven by the same oxygen signal generated in the biofilm interior; the Gac/Rsm cascade and RpoN further integrate nutrient limitation with nitrogen‐fixing biofilm competence [27].

**Figure 1:**
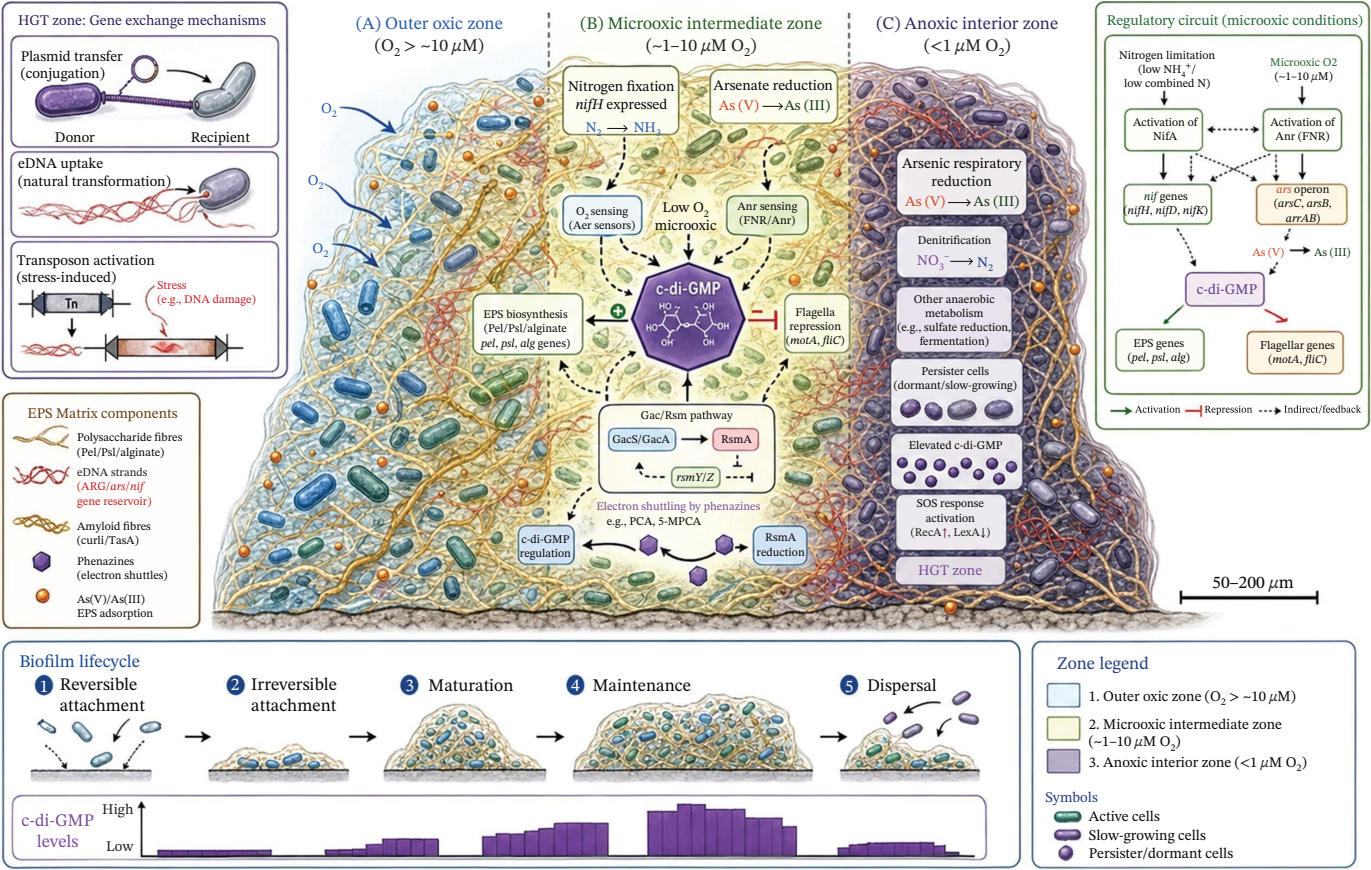
Biofilm as a molecular reactor: spatially stratified regulatory integration of nitrogen fixation, arsenic transformation and antibiotic resistance. Cross‐sectional schematic of a mature biofilm illustrating metabolic and regulatory zonation across the oxygen gradient. The (A) oxic surface zone sustains aerobic respiration, nitrification and As(III) oxidation (AioA/AsoA). The (B) microoxic intermediate zone is the nexus of nitrogenase activity (*nifHDK*, Anr → RpoN/*σ* ^54^ → NifA axis), ArsM‐mediated arsenic methylation and c‐di‐GMP‐driven EPS reinforcement (Gac/Rsm → RsmZ → RsmA → *sadC*/*pslA*). Regulatory modules drawn in the microoxic zone and the NfiS circuit shown in the inset are taken from *P. stutzeri* A1501 and are presented as a worked exemplar of one diazotrophic biofilm regulatory architecture. They are not implied to be conserved across diazotrophs. The (C) anoxic interior harbours persister cells (TA modules, (p)ppGpp) and is shown as permissive for contact‐dependent conjugation and for SOS → intI1 activation. The spatial distribution of gene transfer is drawn here and in Figure 5A, as a single route‐dependent proposed model rather than as a measured maximum, because all available transfer assays are bulk measurements and are spatially blind. EPS components (polysaccharides, eDNA, amyloids and phenazines) provide structural, catalytic and genetic reservoir functions. Inset panels depict the NfiS ncRNA feed‐forward amplification circuit and the five‐stage biofilm lifecycle with corresponding c‐di‐GMP levels.

The defining microenvironmental property of biofilms is steep concentration gradients of oxygen, nutrients, pH, toxicants and metabolites across tens to hundreds of micrometres, arising when metabolic consumption outpaces diffusion through the EPS hydrogel [19]. The oxygen gradient is most consequential (Figure 1A): Aerobic respiration at the biofilm surface produces an oxycline transitioning to fully anoxic conditions within biofilms as thin as 50–100 *μ*m, spatially segregating aerobic respiration, nitrification and As oxidation at the surface; microaerobic denitrification, As(V) reduction and nitrogen fixation at intermediate zones; and fermentation and metabolic dormancy in the anoxic interior [2, 19]. In nitrifying biofilms, ammonia‐oxidising bacteria segregate to the oxygen‐rich surface, whereas anammox bacteria establish at the anaerobic base [46] (Figure 1C); biofilm carriers in IFAS reactors stimulate N_2_O production threefold under low dissolved oxygen conditions compared with activated sludge alone [47]. EPS adsorbs As(V) and As(III) electrostatically, and fluvial biofilms accumulate As(III) extracellularly alongside intracellular DMA^V^ from active methylation—creating As‐selective pressure qualitatively distinct from bulk planktonic exposure [3, 9]. Metabolite crossfeeding across the gradient—lactate and acetate from fermentative interior cells, reduced phenazines from aerobic outer cells and ammonium from nitrogen‐fixing intermediate cells—creates metabolic interdependencies impossible to replicate in planktonic systems [19].

### 2.2. Matrix Architecture and Redox‐Active Moieties: Space for Diazotrophy and AR

The EPS matrix, constituting up to ~90% of biofilm biomass, integrates structural, catalytic and informational functions [48]. Polyanionic exopolysaccharides (Pel/Psl/alginate in *P. aeruginosa*, VPS in *V. cholerae* and cellulose and succinoglycan in soil bacteria) provide viscoelastic scaffolding, sequester cationic antibiotics including aminoglycosides at the biofilm surface, adsorb As species through electrostatic and ligand‐exchange mechanisms, generating a matrix‐associated As pool chemically distinct from the bulk solution [48]. This sorptive concentration must be distinguished from enzymatic transformation, which for the principal ars‐encoded activities is not extracellular. ArsC As(V) reductase is a cytoplasmic enzyme dependent on cytoplasmic glutathione/glutaredoxin or thioredoxin reducing systems, and ArsM As(III) S‐adenosylmethionine methyltransferase is likewise cytoplasmic and dependent on cytoplasmic SAM; both therefore require prior internalisation of As(V) through phosphate transporters (Pst/Pit) or of As(III) through aquaglyceroporins. As(III) oxidase (AioAB/AsoAB) is the exception in being exported by the twin‐arginine translocation system and functioning in the periplasm, coupled to the respiratory chain; it is periplasmic rather than matrix‐retained, and we identified no evidence that it retains catalytic activity while immobilised within the EPS of a defined biofilm. The functional consequence of matrix sorption is therefore to raise the local substrate concentration presented to cell‐associated transporters, to buffer speciation gradients and to retain As against advective loss—not to bypass internalisation. Structural amyloid proteins (curli, TasA and FapC) cooperatively assemble with eDNA into composite networks of exceptional AR, with eDNA nucleating amyloid fibrillation while amyloid fibres protect eDNA from nuclease degradation [49]. eDNA simultaneously functions as structural scaffold, antibiotic sequestrant (enhancing tolerance to tobramycin by ~3‐fold and gentamicin by ~2‐fold in *P. aeruginosa* biofilms) and genetic reservoir enabling HGT at frequencies orders of magnitude above planktonic cultures [22]; As stress‐induced autolysis may simultaneously amplify all three functions for colocated ars, ARG and nif gene‐bearing MGEs. Phenazines in the matrix shuttle electrons between hypoxic interior cells and the oxic zone, sustaining microaerobic metabolisms and feeding back through the phosphodiesterase RmcA to regulate c‐di‐GMP and biofilm morphogenesis [19, 50, 51]; this phenazine‐mediated electron shuttling may also sustain the highly reducing intracellular environment (*E* ^°′^ ≈ −0.42 V) required by nitrogenase [51].

Biofilm stratification creates precisely the conditions enabling simultaneous nitrogen fixation, AR and As transformation. Nitrogenase requires absolute oxygen exclusion—no repair pathway exists for oxidatively inactivated Fe protein—making biofilm interior microenvironments the principal oxygenated settings where sustained diazotrophy is possible [2]. In *Beggiatoa*‐dominated cave biofilms, distinct diazotrophic lineages occupy niche zones defined by sulphide–oxygen counter‐gradients confirmed by nifH transcript analysis [2]. In Rhodopseudomonas sp. BAL398, N_2_ fixation under bulk oxic conditions depends on rosette‐like aggregates in which nitrogenase concentration increases 600‐fold in hypoxic cores [21]. In alga‐colonising Planctomycetota, polysaccharide‐mediated aggregation creates O_2_‐consuming outer layers with microoxic cores, confirming this mechanism across phylogenetically diverse systems [26]. Regulatory coactivation deepens this beyond passive diffusion physics: Anr, RpoN, Gac/Rsm and c‐di‐GMP integrate biofilm commitment with diazotrophic capacity in a unified transcriptional programme in *P. stutzeri* A1501 [27]. For AR, EPS polysaccharides and eDNA sequester drugs at the biofilm surface; intermediate‐zone slow‐growing cells are phenotypically tolerant to antibiotics targeting active biosynthesis; and the innermost anoxic zones harbour persister cells maintained by elevated toxin–antitoxin module activity and starvation‐like conditions [18]. Biofilms as a whole sustain transfer frequencies one to three orders of magnitude above planktonic culture [22]. The assays supporting that figure are, however, bulk measurements, and they do not resolve where within the biofilm transfer occurs. We therefore do not assign a single maximum and instead set out a route‐dependent spatial model as a hypothesis to be tested. Contact‐dependent conjugation should be favoured wherever cell packing and T4SS pilus stabilisation are greatest, which in a mature biofilm includes both the dense interior and the microaerophilic transition zone. Natural transformation should be optimal where competence induction and eDNA release overlap, plausibly close to the oxic–anoxic interface at which autolysis and SOS‐induced lysis are most frequent. Phage transduction should be attenuated in the interior by EPS diffusion limitation and therefore be relatively more important nearer the surface. Outer‐membrane‐vesicle transfer, being contact‐independent, is the least spatially constrained of the four routes. On this reading, the anoxic interior is permissive for conjugation and for SOS‐driven intI1 activation but is not necessarily the site of maximal transfer overall, and the different routes are treated separately rather than being assigned collectively to one zone. The proposed spatial convergence of diazotrophy, transfer‐permissive conditions and persister survival, all organised by a single oxygen‐depletion gradient, would represent a form of mechanistic integration available only in the biofilm state and the deepest justification for treating the biofilm as the essential molecular reactor for understanding the triad.

## 3. Molecular Basis of Nitrogen Fixation Within Biofilms

### 3.1. Nitrogenase Architecture and Oxygen Sensitivity

BNF is catalysed exclusively by nitrogenase, a two‐component metalloenzyme refined over more than 3 billion years of evolution. Three distinct isoenzymes exist—Mo‐nitrogenase (nifHDK), V‐nitrogenase (vnfHDGK) and Fe‐only nitrogenase (anfHDGK)—with Mo‐nitrogenase the most catalytically efficient, phylogenetically widespread and dominant form in environmental communities [52] (Figure 2). All three isoenzymes share the same two‐component architecture and are irreversibly inactivated by molecular oxygen (Figure 2A). The MoFe protein (dinitrogenase; nifD and nifK) is an *α*_2_ *β*_2_ heterotetramer (~230 kDa) harbouring two metal centres per *αβ* unit: the P‐cluster ([8Fe–7S]) serving as an intermediate electron relay and the FeMo‐cofactor ([7Fe–9S–C–Mo–R‐homocitrate])—the active site of N_2_ reduction—buried within the *α* subunit [52]. A central carbide carbon bridges six belt iron atoms flanked by homocitrate and molybdenum; displacement of belt sulphur S2B during CO inhibition studies revealed how a reactive iron site is uncovered during catalysis [53]. Recent cryo‐EM time‐course studies visualised dynamic FeMo‐cofactor rearrangements during substrate reduction, providing the first structural evidence for E0–E3 Lowe–Thorneley intermediate states [54]. The Fe protein (dinitrogenase reductase; nifH) is a homodimer (~64 kDa) bridged by a single [4Fe–4S] cluster with one MgATP‐binding site per subunit [55]. In each catalytic cycle, the MgATP‐bound Fe protein transiently associates with the MoFe protein, transfers a single electron and dissociates upon hydrolysis of two MgATP—the overall rate‐limiting step—repeated a minimum of eight times per N_2_, consuming at least 16 MgATP and yielding 2 NH_3_ + H_2_ [55]. Kinetic analyses indicate the actual cost approaches 20–25 MgATP per N_2_ because of unproductive electron transfer cycles [56].

**Figure 2:**
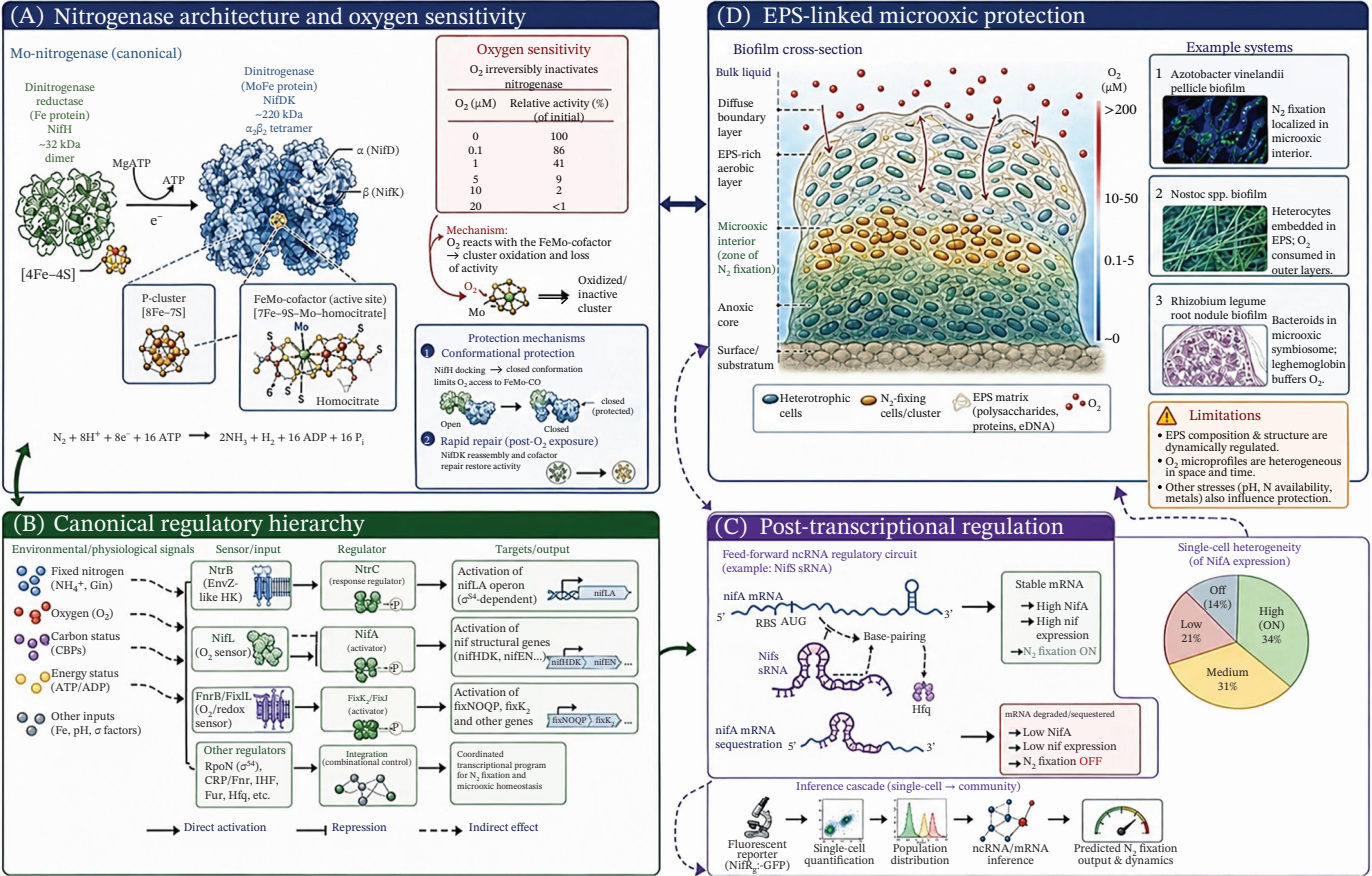
Molecular basis of nitrogen fixation within biofilms. (A) Mo‐nitrogenase two‐component architecture (MoFe/Fe proteins), metal cluster identity, catalytic stoichiometry (≥ 16 MgATP/N₂) and asymmetric O₂ inactivation countered by respiratory (FeSII conformational protection) and community‐level (biofilm oxycline) mechanisms. (B) Multi‐input regulatory logic gate: NifL‐FAD redox, PII‐mediated N‐status sensing (GlnB/GlnK uridylylation) and ATP/ADP ratio converge on NifA–RpoN(*σ* ^54^) to gate nifHDK expression; DraT/DraG add post‐translational modulation. (C) NifS/NifR sRNA feed‐forward amplification loop and spatial heterogeneity of nifH expression (~11% active cells). (D) EPS‐driven oxycline, Anr‐mediated O₂ sensing and critical nifH inferential limitations.

Oxygen sensitivity is extreme and asymmetric: The Fe protein has a half‐life of ~30–45 s in air, whereas the MoFe protein survives ~10 min before irreversible metal cluster oxidative destruction [55]. No repair mechanism for O_2_‐inactivated nitrogenase exists, making de novo biosynthesis mandatory for activity recovery. Two cellular mechanisms mitigate this liability. Respiratory protection, classically characterised in *Azotobacter* species, depletes O_2_ at the cell surface through highly uncoupled terminal oxidases with exceptional O_2_ affinity; *A. vinelandii* sustains diazotrophy at dissolved O_2_ up to 230 *μ*M through this mechanism [57]. Conformational protection operates when respiratory protection is overwhelmed: The [2Fe–2S] ferredoxin FeSII (Shethna protein II) is oxidised by O_2_, undergoes a domain‐swap conformational change and associates with the Fe protein to form a protective ternary complex; a landmark cryo‐EM study resolved this as a 620 kDa core that further polymerises into large filamentous structures that are catalytically inactive but rapidly reactivatable upon O_2_ depletion [58]. In the biofilm context, cells in the microoxic interior may cycle dynamically between active nitrogen fixation and the conformationally protected state in response to O_2_ fluctuations driven by respiratory activity or physical biofilm perturbation—a dynamic regulation mechanism uncharacterised at single‐cell or spatial resolution in any biofilm system. At the community scale, the outer aerobic biofilm layers collectively function as a distributed respiratory protection apparatus for interior diazotrophs, consuming O_2_ through growth‐associated metabolism and passively maintaining the microoxic conditions required for nitrogenase—thermodynamically more robust than single‐cell respiratory protection, which is constrained by individual carbon availability.

### 3.2. Canonical Regulatory Hierarchy: NifA/NifL, RpoN, PII, Ammonium Repression and Redox Control

Expression of nitrogenase structural genes (nifHDK) and accessory genes for FeMo‐cofactor biosynthesis (nifBENQXUSW), electron transport and regulation is under multilevel transcriptional and post‐transcriptional control integrating signals for O_2_ status, fixed‐nitrogen availability, carbon status and energy charge (Figure 2B) [59–61]. The conserved core is the NtrC/NifA/RpoN cascade, in which global nitrogen status hierarchically controls all nif promoters. NifA is the master transcriptional activator of all nif genes—a *σ* ^54^‐dependent enhancer‐binding protein (EBP) with three functional domains: an N‐terminal GAF domain binding 2‐oxoglutarate (2‐OG) as a C/N ratio surrogate; a central AAA+ ATPase motor domain driving open‐complex formation at −24/−12 *σ* ^54^‐dependent nif promoters; and a C‐terminal helix‐turn‐helix domain recognising upstream activation sequences [59, 62]. NifL, a FAD‐containing antiactivator in organisms including *A. vinelandii* and *Herbaspirillum seropedicae*, provides strict negative regulation: Oxidised FAD under oxic conditions drives inhibitory binding to NifA′s AAA+ domain to block ATPase activity, whereas reduced FAD under anoxic conditions releases NifA for nif activation [62, 63]. NifL additionally senses energy status through ADP binding, which overrides the redox signal to maintain NifA inhibition when ATP supply is insufficient regardless of O_2_ level. A recent low‐resolution cryo‐EM structure of the NifL–NifA complex mapped the interaction surface for the first time, revealing that NifL′s GHKL and DH domains sterically block NifA′s AAA+ domain—opening routes to engineering diazotrophs with derepressed nitrogen fixation for agricultural applications [64].

The PII proteins GlnB and GlnK serve as the primary intracellular integrators of nitrogen status, simultaneously sensing ATP/ADP ratio, 2‐OG concentration and GlnD‐mediated uridylylation state to relay nitrogen signals to NifL and the NtrB/NtrC system controlling nifA expression [65]. Under nitrogen limitation, declining glutamine/2‐OG causes GlnD to uridylylate both proteins: GlnB‐UMP activates NtrB, which phosphorylates NtrC to induce nifA and glnA transcription; simultaneously, GlnK‐UMP is sequestered to inner membrane ammonium transporter AmtB, preventing GlnK from stimulating NifL′s inhibitory function and permitting NifA activity [65]. Under nitrogen sufficiency, deuridylylated GlnK restimulates NifL inhibition of NifA, rapidly shutting down nif expression. The integrated outcome is a multi‐input logic gate: Nitrogenase genes are expressed only when O_2_ is low (reduced NifL‐FAD), nitrogen is limiting (GlnK‐UMP sequestered from NifL) and energy is adequate (low ADP/ATP). Post‐translational control adds a final layer: In photosynthetic and some aerobic diazotrophs, DraT immediately inactivates the Fe protein through covalent ADP‐ribosylation of a conserved Arg residue in response to ammonium addition or energy limitation, with DraG reversing this upon nitrogen‐limiting conditions [59]. In soil diazotrophs lacking the DraT/DraG system, ammonium repression operates principally through the NifL/PII transcriptional axis.

### 3.3. Post‐Transcriptional Regulation, Expression Heterogeneity and Transcript Stability

Transcriptional activation of nif genes by the NifA/RpoN cascade is necessary but not sufficient for sustained nitrogen fixation competence. Multiple post‐transcriptional circuits modulate nitrogenase expression and activity in response to the fluctuating O_2_ levels and nutritional states experienced by cells within the biofilm, substantially amplifying or attenuating the transcriptional output of the NifA/RpoN system (Figure 2C). A paradigmatic example is the small non‐coding RNA NfiS in *P. stutzeri* A1501, induced specifically under nitrogen fixation conditions and osmotic stress through the RpoN/NtrC/NifA cascade [29]. Through a stem‐loop structure containing an 11‐nucleotide seed region, NfiS base‐pairs with the 5 ^′^‐UTR of nifK mRNA, increasing translation efficiency and mRNA half‐life to enhance nitrogenase activity approximately twofold above the level achievable by transcriptional activation alone—creating a feed‐forward amplification loop in which NifA drives both nitrogenase structural gene transcription and the NfiS stabiliser protecting those transcripts [29]. Critically, NfiS performs a dual regulatory function; it simultaneously targets katB mRNA encoding a catalase–peroxidase, upregulating oxidative stress protection at the post‐transcriptional level [30]. This dual targeting directly connects the nitrogen fixation programme to antioxidant defence—particularly relevant in biofilm microoxic zones where nitrogenase‐active cells are exposed to reactive oxygen species generated by neighbouring aerobic cells or redox‐active matrix metabolites. A companion ncRNA, NfiR, extends this regulatory layer to additional targets including iron acquisition and hydrogen evolution—functions directly relevant to nitrogenase electron supply and metal cofactor availability [28].

Single‐cell nifH expression within morphologically structured communities is profoundly heterogeneous. In an aerobic *Gluconacetobacter*‐related endophyte of *Populus* trees (strain WPB), only ~11% of cells in genetically identical colonies express nifH at any given time, and cell association exerted a far stronger influence on nifH expression (*β* = −3.32, *p* < 0.0001) than nitrogen status alone [66]. This indicates that microenvironmental or cell‐contact signals generated by aggregation—not bulk nutrient availability—are the primary determinants of which cells become diazotrophically active. In the biofilm context, this implies that a cell′s spatial position, which determines its local O_2_, nutrient and signalling environment, is the principal driver of nifH expression, with profound implications for interpreting community‐level nitrogen fixation rates from bulk transcript measurements. Transcript stability and enzyme‐level regulation further decouple nifH transcript abundance from nitrogenase activity. Upon O_2_ exposure, the Fe protein is irreversibly inactivated within seconds before nifH mRNA levels are measurably affected; DraT‐mediated ADP‐ribosylation inactivates the Fe protein within minutes of ammonium sensing without changes in transcript levels. These considerations establish that simultaneous measurement of nifH transcripts, nitrogenase protein abundance, Fe protein modification state and direct N_2_ fixation rates—via ^15^N_2_ incorporation with NanoSIMS or acetylene reduction with spatial resolution—is required to characterise nitrogen fixation in biofilm systems quantitatively.

### 3.4. EPS‐Linked Microoxic Protection, Aerobic Diazotrophy and nifH‐Centric Dynamics

EPS composition, matrix thickness and community stratification are direct determinants of nitrogen fixation capacity at the community level (Figure 2D). The polysaccharide hydrogel retards O_2_ penetration—O_2_ diffusivity in hydrated biofilm EPS is approximately 80% of that in water—and, combined with active O_2_ consumption throughout the embedded community, produces the steep oxyclines supporting interior microoxic nitrogenase activity [19]. Beyond this physical barrier, the regulatory coupling between EPS production and nif gene expression ensures that the same signal inputs driving matrix biosynthesis—nitrogen limitation, surface contact and microoxic O_2_—simultaneously activate nitrogen fixation. In *P. stutzeri* A1501, the Anr transcription factor senses declining O_2_ through its oxygen‐labile [4Fe–4S] cluster; under microoxic conditions generated in the biofilm interior, active Anr upregulates nifH expression, narG/L (nitrate reductase) and RpoS expression, directly coupling the biofilm developmental programme to nitrogenase activation through a shared O_2_‐sensing regulator [28]. Disruption of anr not only impaired nifH and nifC expression (confirmed by qRT‐PCR) but also reduced biofilm formation, demonstrating bidirectional dependency between the Anr‐regulated oxygen response and nitrogen‐fixing biofilm competence [28].

The nifH gene has been the canonical molecular marker for diazotrophs in environmental surveys for over three decades, but its use for mechanistic inference in biofilm systems carries limitations requiring critical engagement. First, primer design introduces systematic bias: Evaluation of 51 universal and 35 group‐specific nifH primers showed that although 15 universal primers amplify ≥ 90% of database sequences, 23 commonly used primers amplify less than 50%, generating strongly primer‐dependent community profiles [67]. Many primers additionally coamplify paralogs in phylogenetic Clusters II and IV—including bchL/chlL (protochlorophyllide reductase) and pdt‐like sequences that do not catalyse N_2_ reduction—systematically overestimating diazotroph diversity when amplicon libraries are not screened for nontarget sequences [67]. Single‐base primer mismatches cause systematic underdetection of ecologically important clades including Gammaproteobacteria and Planctomycetota [68].

Second, nifH gene presence does not guarantee functional nitrogenase capacity. Analysis of 3500+ nitrogen‐fixing genomes found that 323 of 1352 putative diazotrophs identified by nifH presence lacked the complete nifHDK cluster required for functional enzyme assembly, meaning nifH presence at 8.4% of total surveyed genomes overestimates proven N_2_ fixation capability [69]. Many organisms carry nifH as an orphan gene without adjacent nifD, nifK or the accessory genes required for FeMo‐cofactor biosynthesis. Third, a six‐step inferential cascade separates nifH gene detection from confirmed nitrogen fixation: (i) gene presence → (ii) nifH transcription → (iii) Fe protein translation → (iv) functional holoenzyme assembly with metalloclusters → (v) protection from O_2_ inactivation → (vi) active N_2_ reduction. Each step can be independently disrupted by post‐translational modification, metal limitation, redox state, O_2_ exposure or cofactor biosynthesis bottlenecks without affecting the preceding step. In biofilm‐specific studies requiring causal mechanistic inference, nifH amplicon data must be complemented with spatial transcript assays (FISH‐based methods), targeted proteomics for nitrogenase protein detection and direct N_2_ fixation measurements using ^15^N_2_ isotope methods combined with NanoSIMS or BioOrthogonal Non‐Canonical Amino acid Tagging (BONCAT).

## 4. Signalling Networks Connecting Sessility and Diazotrophy

### 4.1. Quorum Sensing (QS) in Diazotrophs and Rhizobia

QS, the cell density‐dependent regulation of gene expression through accumulation of diffusible autoinducers, links population size and community architecture to cooperative behaviours including EPS production, biofilm formation and symbiotic nitrogen fixation (Figure 3). In most Gram‐negative diazotrophs and rhizobia, the dominant system is LuxI/LuxR‐type: A LuxI‐homologue synthesises N‐acyl‐homoserine lactones (AHLs) that freely diffuse and accumulate proportionally to cell density; above threshold concentrations, AHL–LuxR complexes activate QS‐regulated target genes (Figure 3A) [31]. In rhizobia, QS is mechanistically integrated into symbiotic nitrogen fixation. *Rhizobium leguminosarum* bv. *viciae* carries a hierarchical multitiered cascade comprising CinI/CinR, RaiI/RaiR and BisI/BisR systems, in which high‐concentration AHL signals from CinI/CinR activate RaiI/RaiR at a lower threshold, tuning density sensing to different stages of host colonisation and nodule development [31]. In *Sinorhizobium meliloti*, the SinI/SinR and ExpR/ExpI systems regulate EPS II (galactoglucan) biosynthesis; the specific structural features of EPS II are recognised by the plant host as compatible signals for infection thread development and progression to nitrogen‐fixing symbiosomes and QS‐deficient mutants show aberrant infection threads with reduced nitrogen fixation efficiency [31]. In free‐living diazotrophs, AHL QS similarly links population density to nitrogen‐fixing biofilm competence: In *Azospirillum brasilense*, AHL production regulates flocculation, EPS production and root colonisation efficiency, underpinning the formation of diazotrophically active biofilm communities on root surfaces [70]. In wastewater treatment biofilm reactors, specific AHL molecules (C_6_‐HSL, C_8_‐HSL) regulate EPS production and biofilm architecture, which in turn determine the O_2_ gradient structure controlling the balance between nitrification, denitrification and nitrogen fixation [71, 72]. This creates a unified density‐sensing pathway in which the same autoinducer signal promoting EPS matrix production also coordinates the metabolic activities dependent on the microenvironmental conditions that matrix generates. Critical mechanistic questions remain unresolved: whether AHL QS directly regulates nif gene expression in free‐living diazotrophs; whether the O_2_ gradient created by QS‐regulated EPS production feeds back to activate Anr or NifL, coupling population density to nitrogenase gene expression; and whether As stress alters AHL production or degradation in diazotrophic biofilms, indirectly affecting nitrogen fixation capacity. These are mechanistically tractable and constitute high‐priority targets for future investigation.

**Figure 3:**
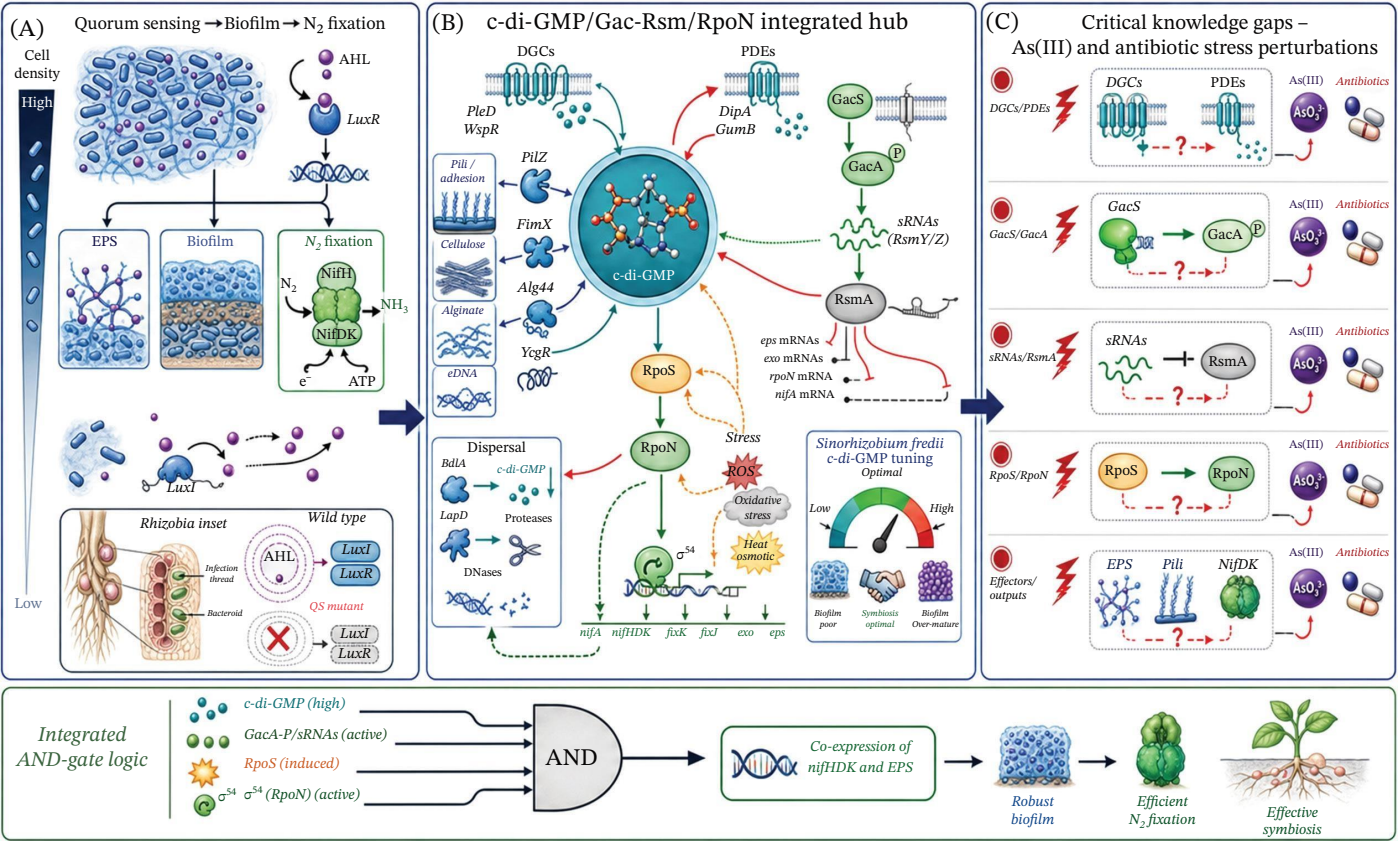
Signalling networks connecting sessility and diazotrophy. (A) LuxI/LuxR‐type AHL quorum sensing links cell density to EPS production, biofilm‐generated O₂ gradients and symbiotic nitrogen fixation efficiency in free‐living diazotrophs and rhizobia (hierarchical CinI/CinR→RaiI/RaiR cascade; SinI/SinR‐mediated EPS II for symbiosome formation). (B) Integrated central hub in *P. stutzeri* A1501: Gac/Rsm cascade sequesters RsmA to coderepress *sadC* (c‐di‐GMP↑) and *pslA* (EPS); RpoN(*σ* ^54^) coactivates *pslA* and *nifA* from shared promoters, with RpoS providing a temporal braking mechanism. (C) Five mechanistically tractable but unresolved knowledge gaps: As(III)‐mediated c‐di‐GMP dysregulation, SOS‐driven *intI1* mobilisation, ArsR regulon interference with Gac/Rsm, 2‐OG pool perturbation of PII/NifL and As‐induced EPS remodelling altering microoxic nifH‐active zones.

### 4.2. C‐di‐GMP: Master Regulator Linking Surface Commitment, Matrix Production and Diazotrophic Activation

C‐di‐GMP governs the binary decision between motile planktonic and sessile biofilm lifestyles, and mounting evidence establishes that this same molecule interfaces directly with the nitrogen fixation regulatory network in diazotrophic bacteria (Figure 3B) [23]. In *P. stutzeri* A1501, c‐di‐GMP metabolism is integrated into the Gac/Rsm–RpoN network coregulating EPS production and nitrogen fixation: Diguanylate cyclase SadC synthesises c‐di‐GMP to promote Psl polysaccharide production via pslA mRNA derepression, whereas phosphodiesterase BifA degrades c‐di‐GMP to promote dispersal; both sadC and pslA mRNAs are post‐transcriptionally controlled by the translational repressor RsmA [27]. This coupling means that the Gac/Rsm signal driving EPS production simultaneously elevates c‐di‐GMP by releasing sadC mRNA from RsmA repression, creating a positive feedback loop reinforcing surface commitment and matrix maturation. Critically, RpoN activates both nifA (nitrogen fixation master activator) and pslA (EPS biosynthesis) through shared *σ* ^54^‐dependent promoters under nitrogen‐limiting microoxic conditions—precisely the conditions generated in biofilm interiors—architecturally cowiring nitrogen fixation activation with biofilm matrix reinforcement through shared dependence on a single sigma factor [27]. Disruption of rpoN in *P. stutzeri* A1501 simultaneously eliminated nitrogenase activity and severely impaired biofilm formation and EPS production, providing genetic proof of this coregulation [27].

In *Sinorhizobium fredii*, elevated c‐di‐GMP increased autoaggregation and altered EPS composition—including fucose content of exopolysaccharide chains serving as plant‐recognisable symbiotic signals—promoting biofilm formation while impairing initial root colonisation at specific c‐di‐GMP levels, revealing that rhizobia must fine‐tune c‐di‐GMP to balance surface adhesion, EPS secretion and colonisation competence required for functional symbiotic nitrogen fixation [73]. High numbers of GGDEF‐EAL domain proteins encoded by rhizobia reflect an extensive c‐di‐GMP signalling repertoire modulating species‐specific symbiotic pathways across lineages [74, 75].

The intersection of c‐di‐GMP signalling with As stress represents a critical knowledge gap. As(III) alters EPS production and biofilm formation in exposed strains [7, 8], suggesting perturbation of c‐di‐GMP metabolism, though the molecular mechanism—whether through direct effects on DGC/PDE enzyme activities, oxidative modification of sensing domains or indirect effects through altered redox potential or ATP availability—remains unestablished. Given that DGC PAS domain sensing is redox‐sensitive in some systems and As stress elevates cellular ROS and modifies intracellular redox state, As‐induced c‐di‐GMP perturbations could alter both biofilm architecture and nitrogen fixation capacity through the RpoN‐linked coregulatory axis. Some of the major regulatory modules are listed in Table 2.

**Table 2:** Reported occurrence and experimental support for the major regulatory modules across representative diazotrophs.

Regulatory module	*P. stutzeri* A1501	*A. vinelandii*	*Klebsiella oxytoca*/*K. pneumoniae*	*A. brasilense*	*Herbaspirillum seropedicae*	*Rhizobium*/*Sinorhizobium* spp.
NifA activator with RpoN (*σ* ^54^)	✓	✓	✓	✓	✓	✓
NifL redox/2‐OG antiactivation of NifA	✓	✓	✓	—	✓	– (FixL/FixJ–FixK and O_2_‐labile NifA instead)
PII proteins (GlnB/GlnK) as N‐status integrators	✓	✓	✓	✓	✓	✓
DraT/DraG post‐translational Fe‐protein modification†	n.d.	—	—	✓	n.d.	—
FeSII (‘Shethna’) conformational O_2_ protection†	n.d.	✓	—	n.d.	n.d.	n.d.
Anr/FNR‐type O_2_ sensing coupled to nif expression†	✓	(✓)	n.d.	n.d.	n.d.	—
Gac/Rsm–RsmA/RsmZ post‐transcriptional control of EPS†	✓	✓	n.d.	n.d.	n.d.	n.d.
NfiS/NfiR regulatory ncRNA stabilising nifK and katB†	✓	—	—	—	—	—
c‐di‐GMP (DGC/PDE) control of matrix production	✓	(✓)	(✓)	(✓)	n.d.	✓ (*S. fredii*)
AHL quorum sensing coupled to EPS and symbiotic fixation	n.d.	n.d.	n.d.	✓	n.d.	✓ (CinI/CinR–RaiI/RaiR; SinI/SinR; ExpR/ExpI)
ars regulon (arsR, arsC, arsB/acr3, arsM) present	✓	(✓)	✓	(✓)	(✓)	✓ (AqpS adjacent to symbiotic cluster in *S. meliloti*)

*Note:* [✓], reported with direct experimental support in that organism; [(✓)], inferred from genome content or from a close relative without direct functional demonstration; [—], not reported in that organism, which should not be read as demonstrated absence; n.d., not determined. Modules marked [†] are, on current evidence, restricted to the organism or lineage indicated and are therefore treated in this review as exemplars rather than as conserved features of diazotrophic biofilms.

### 4.3. The Gac/Rsm Pathway: Post‐Transcriptional Integration of Carbon Status, Biofilm Formation and Diazotrophy

The Gac/Rsm pathway is the most thoroughly characterised molecular link between nutritional status, biofilm formation and nitrogen fixation in plant‐associated diazotrophs, operating through a four‐node relay (Figure 3B) [27]. Its architecture is set out below as it has been established in *P. stutzeri* A1501 and, for the upstream components, in nondiazotrophic pseudomonads. It should not be assumed to operate in phylogenetically distant diazotrophs: The NifA–RpoN core and PII‐mediated nitrogen sensing are broadly conserved, whereas Gac/Rsm coupling, the NfiS ncRNA, Anr‐type oxygen sensing, FeSII‐mediated conformational protection and DraT/DraG post‐translational control are each restricted to particular lineages. Sensor histidine kinase GacS phosphorylates response regulator GacA, which activates transcription of regulatory ncRNAs RsmZ (and paralogues RsmY and RsmX) carrying multiple GGA motifs in exposed stem loops. These motifs allow RsmZ/Y to competitively sequester RsmA (CsrA in enterobacteria)—a global post‐transcriptional repressor that binds 5 ^′^‐UTR GGA motifs to block ribosome access—thereby derepressing translation of multiple biofilm‐promoting genes simultaneously. In *P. stutzeri* A1501, the critical RsmA targets are pslA (Psl polysaccharide initiating glycosyltransferase) and sadC (diguanylate cyclase synthesising c‐di‐GMP): RsmZ sequestration of RsmA concurrently derepresses both, increasing EPS matrix development and elevating c‐di‐GMP approximately twofold to reinforce surface commitment [27]. RsmZ thus functions as a signal amplifier in which a small change in GacS/GacA activation state produces a large coordinated change in EPS and c‐di‐GMP through concurrent positive feedback derepression, with ddPCR‐based absolute transcript quantification confirming that RsmZ abundance is the critical switching variable that increases dramatically during biofilm maturation.

The RpoS sigma factor (*σ* ^s^) provides stage‐specific negative feedback. RpoS transcripts are very low in early‐stage *P. stutzeri* A1501 biofilm (12 h) but increase approximately sevenfold in mature biofilm (48 h), where rising RpoS levels downregulate RsmZ expression through a GacA‐independent indirect mechanism, releasing RsmA from sequestration and progressively suppressing sadC and pslA translation—a molecular braking mechanism limiting unlimited biofilm expansion and priming dispersal [27]. A reciprocal relationship completes the circuit: RsmA directly represses rpoS mRNA translation through the conserved 5 ^′^‐UTR GGA motif (confirmed by micro‐scale thermophoresis binding assays), suppressing RpoS accumulation in early‐stage biofilm when high RsmA activity is required for productive initiation. This mutually repressive RsmA–RpoS relationship constitutes a developmental timing device for biofilm stage transitions. The nitrogen fixation regulatory network is superimposed on this Gac/Rsm/RpoS scaffold through the shared RpoN sigma factor and NfiS ncRNA cascade. RpoN (*σ* ^54^) activates both pslA (EPS) and nifA (nitrogen fixation master regulator) from adjacent *σ* ^54^‐dependent promoters; NifA then activates the entire nif cluster and nfiS transcription, which post‐transcriptionally stabilises nifK and katB mRNAs [29]. The resulting regulatory logic is a coherent feed‐forward programme: nitrogen limitation and microoxic conditions in the biofilm interior → RpoN activation → simultaneous EPS reinforcement (pslA) + nitrogenase activation (nifA) + NfiS‐mediated mRNA stabilisation. This coinduction is more robust than sequential induction because EPS production and nitrogenase biosynthesis are activated in the same cells by the same inducing signals, ensuring the structural microenvironment (EPS‐mediated O_2_ depletion) and the biochemical activity (nitrogenase) are produced together.

### 4.4. Open Questions on Signal Integration Under As and Antibiotic Stress

The impact of As stress and antibiotic exposure on this integrated regulatory network is critical and almost entirely unexplored (Figure 3C). Five mechanistic priorities can be defined.

First, does As stress rewire c‐di‐GMP signalling in diazotrophic biofilms? As(III) is a thiol‐reactive metalloid modifying cysteine residues in sensor histidine kinases and redox‐sensing regulators, and As‐induced ROS could modify PAS domain sensor cysteines in DGC/PDE proteins, potentially altering c‐di‐GMP levels and disrupting the RpoN‐co‐regulated EPS/nif axis. To our knowledge and within the scope of the search described at the end of Section 1, we did not identify a study in which intracellular c‐di‐GMP was directly quantified in an As‐stressed diazotroph.

Second, does antibiotic‐induced SOS response alter nitrogen fixation or biofilm regulatory gene expression? SOS induction by quinolones promotes error‐prone DNA polymerase activity and MGE mobility—increasing mutation rates in nif genes and ARGs—and is associated with altered biofilm morphology in *P. aeruginosa*, but whether analogous interactions affect GacS/GacA or RpoN axes in diazotrophs is entirely unknown.

Third, does As alter RsmA/RsmZ stoichiometry or Gac/Rsm circuit expression? ArsR proteins are winged‐helix transcription factors that could potentially bind promoters beyond canonical ars genes, raising the possibility of direct transcriptional interference with Gac/Rsm components. We identified no genome‐wide mapping of an ArsR regulon in any diazotroph grown under nitrogen‐fixing conditions within the literature searched.

Fourth, does As‐induced perturbation of cellular metabolism alter the intracellular 2‐OG pool, thereby indirectly modulating NifL–NifA behaviour through the PII‐GlnB sensing axis? This represents a plausible but entirely untested mechanistic link between As stress and nitrogen fixation regulation.

Fifth, how does As‐induced EPS remodelling alter the O_2_ diffusion profile and spatial distribution of nifH‐expressing cells? As affects EPS composition and quantity in exposed biofilms [7, 8]; thicker or more densely crosslinked As‐stressed EPS could deepen the microoxic zone and expand nitrogenase‐active volume, whereas disrupted matrix structure could increase O_2_ permeability and collapse the protective microoxic zone. Resolving this requires O_2_ microsensor profiling of As‐exposed diazotrophic biofilms combined with spatially resolved nifH mRNA detection—currently feasible but unreported.

## 5. Biofilm: An Interactive Matrix for As‐N Dynamics and Dissemination of AR

### 5.1. As Chemistry and Microbial Response Inside Biofilms

As occurs in four oxidation states, but inorganic As(III) and As(V) dominate bacterial exposure: As(V) prevails under oxic conditions, whereas the more mobile and toxic As(III) accumulates in reducing biofilm interiors and flooded sediments [4, 76]. Microbial ArsM methyltransferases generate MMA(V/III), DMA(V/III) and TMAO via SAM‐dependent methylation; the trivalent intermediates MMA(III) and DMA(III) are more toxic than inorganic As(III) due to enhanced thiol reactivity, so methylation imposes a second organic tier of As stress alongside detoxification [77–79]. As(V) enters cells via phosphate transporters (Pst and Pit), and under phosphate‐limited biofilm interiors, this competition favours As(V) uptake [76]. As(III) enters as As(OH)_3_ through aquaglyceroporins (GlpF and AqpS), whereas methylarsenicals traverse AqpS and the ArsP permease [80, 81]. The EPS matrix concentrates and transforms As: EPS‐bearing biofilms adsorb mainly As(V) through polysaccharide–protein interactions, and after EPS removal, As(III) dominates, whereas methylated arsenicals disappear, demonstrating that EPS drives retention, oxidation and methylation [82–84]. Intracellular DMA(V) enrichment confirms active transformation as a sessile‐state detoxification strategy [85]. Critically, arsM expression and aquaglyceroporin‐mediated As(III) uptake are suppressed > 80% at 250 mg C/L DOC, with glucose, DOM and leaf leachate causing carbon catabolite repression at rhizosphere‐relevant concentrations [86].

As resistance is encoded by modular ars operons in nearly every sequenced bacterium [87]. The dimeric SmtB/ArsR repressor coordinates As(III) at an *α*3N Cys_3_ site (Kd ~10^−8^ M), derepressing transcription ~10^4^‐fold [33]. ArsC reduces As(V) to As(III) using GSH/glutaredoxin or thioredoxin, supplemented by abiotic reduction in reducing interiors [76, 85]. ArsB and Acr3 efflux As(III) (with ArsA forming an ATP‐driven pump), imposing metabolic costs that compete with nitrogen fixation [87]. ArsM methylates As(III) through MMA(III) and DMA(III) to volatile TMA, with MMA(III) acting as a primitive As antibiotic against non‐resistant competitors [77]; diazotrophy‐derived ammonium stimulates SAM synthesis, coupling N_2_ fixation to methylation. A secondary tier—ArsH (FMN oxidoreductase), ArsI (C–As lyase) and ArsP (MFS permease)—handles organoarsenic metabolites, with accessory ArsD, ArsN, ArsJ and AioA/AioB enabling community‐level detoxification [76, 88].

The ArsR response is global. In *Agrobacterium tumefaciens* 5A, four ArsR paralogues form a hierarchical cascade (ArsR1 → ArsR4 → ArsR2 → ArsR3) regulating a stimulon covering As resistance, phosphate acquisition, carbon catabolism, chemotaxis, copper tolerance and iron homeostasis; *arsR3* deletion alone perturbed 34 iron acquisition genes [33]. As(III) disrupts pre‐existing Fe–S clusters in aconitase, succinate dehydrogenase and nitrogenase Fe protein by displacing iron, triggering Fenton chemistry and ROS, and compensatory upregulation of Isc and SUF assembly machinery is ArsR‐independent [89]. As(III) binding also shifts ArsR3 to a non‐DNA‐binding state, suppressing iron uptake [33]. Phosphate limitation in biofilm interiors activates the PhoRB–AioSR axis, coupling nutritional microenvironment to As(III) oxidase expression.

EPS contributes uniquely biofilm‐specific sequestration. FTIR spectra show multidentate As coordination to O–H, N–H, C=O and P–O groups [83, 84, 90]. Redox stratification drives speciation: Extracellular As(III) accumulates despite ambient As(V) dominance, whereas DMA(V) concentrates intracellularly for export [85]. In anoxic sediments, Arr‐type respiratory As(V) reduction couples to organic carbon oxidation, and methanogen–bacterial syntrophy amplifies methylation in paddy and wetland biofilms [91]. Carbon catabolite repression predicts an interior‐to‐exterior arsM expression gradient inverse to oxygen, awaiting NanoSIMS‐level confirmation [86].

### 5.2. How As Perturbs or Selects Nitrogen‐Fixing Biofilms

The As–N_2_ fixation relationship is bidirectional, dependent on concentration, speciation, diazotroph identity, nitrogen status and structural context. Because most studies report proxy endpoints (nifH abundance, community composition and soil N indices) rather than direct ^15^N_2_ rates, mechanistic inferences remain provisional. At the isolate level, As(III) at 50–500 *μ*M significantly suppressed nifH transcripts in *β*‐, *γ*‐Proteobacterial and Firmicute rice‐field diazotrophs (77.69% PCA variance) and concurrently reduced EPS production [32]. Because nifH and EPS biosynthesis share RpoN and Gac/Rsm regulation, this cosuppression points to upstream interference rather than parallel targeting. At the community level in As/Sb‐contaminated mining soils, contamination raised nifH alpha diversity and abundance yet weakened co‐occurrence network strength and shifted composition towards As‐adapted taxa [71, 72]; generalists showed beta‐diversity shifts, whereas specialists remained compositionally stable, with both displaced from central network positions [92]. Elevated diversity therefore decouples from functional capacity. In Hetao Plain groundwaters, ^15^N_2_ tracing measured fixation at 9.5–585.4 nmol N L^−1^ h^−1^ by *Stutzerimonas*, *Pseudomonas*, *Paraburkholderia* and *Klebsiella*, with nifH correlated to O_2_ and Fe(II) rather than As, implying shared redox control [93].

Hyperaccumulator rhizospheres provide the clearest case of As‐stimulated fixation. In *Pteris vittata*, As‐enriched *Rhizobium*, *Mesorhizobium*, *Desulfovibrio* and Cyanobacteria; elevated total N, NH_4_^+^ and NO_3_^−^; and root‐secreted As‐related metabolites selectively recruited As‐tolerant diazotrophs—rendering As an active chemical signal [9]. Homogeneous selection rose from 41.6% to 54.9% along the contamination gradient, convergently selecting a 42‐ASV core assembly module [34]. In *V. zizanioides*, arsM‐carrying and nifD/nifK‐bearing organisms coenriched under As exposure, with methylation and fixation rates positively correlated in root compartments but not bulk soil [10]—consistent with ammonium‐driven SAM provisioning fuelling ArsM. In paddy soils, As impairs ammonia‐oxidisers, redirects N cycling towards denitrification and coenriches ARGs with ars genes [74, 75]. Flooded paddies thus concentrate the As–diazotrophy–resistance triad.

Five mechanisms plausibly link As to nitrogenase impairment.

1. Oxidative stress: As(III) oxidation, NADPH oxidase activation (~60% of As‐induced ROS), and respiratory chain disturbance generate O_2_^−^, H_2_O_2_ and •OH [94]; •OH inactivates Fe protein, and H_2_O_2_ oxidises NifL FAD to repress NifA [33]. The NfiS‐driven katB upregulation in *P. stutzeri* A1501 [30] constitutes a pre‐emptive antioxidant buffer.
2. Thiol‐mediated Fe–S disruption: As(III) binds protein thiols (Kd 10^−8^–10^−10^ M), targets NifH cluster cysteines and inhibits IscS, starving NifH/NifDK/NifU/NifS/NifB assembly; compensatory Isc/SUF upregulation is itself costly ([89]).
3. ATP burden: ArsAB efflux (≥ 2 ATP/As), ArsC–GSH/NADPH demand and ArsM SAM consumption [87] compete with the ≥ 16 ATP per N_2_ required for fixation.
4. Aquaglyceroporin coupling: GlpF upregulation under N‐fixing conditions raises As(III) influx alongside carbon uptake.
5. Carbon/redox reallocation: As‐stimulated TCA upregulation [89] diverts carbon from EPS and nif expression, potentially dropping C/N below NifA activation thresholds.

Direct mechanistic evidence at the protein level is essentially absent in the literature searched. We identified no study in which the following were measured simultaneously: nitrogenase‐specific activity, DraT‐mediated Fe protein ADP‐ribosylation, Fe–S cluster integrity (EPR) and nifH transcripts in As‐exposed biofilms. The ArsR regulon has not been mapped in any diazotroph under N‐fixing conditions—the Rawle et al. [33] stimulon was N‐sufficient—and direct ArsR binding to nif or Gac/Rsm promoters remains untested. Spatial questions also persist: Outer oxic As‐rich zones may face dual inhibition, whereas inner microoxic reduced‐As zones may protect nitrogenase, requiring depth‐resolved microsensor, NanoSIMS and spatial‐transcriptomic mapping to resolve.

### 5.3. Molecular Routes to AR Development in Biofilms

Three mechanistically distinct phenomena operate simultaneously in biofilms. Inherited resistance is a heritable, mutation‐ or HGT‐based MIC shift stable after antibiotic removal [95, 96]. Tolerance is reversible, condition‐dependent survival of bactericidal exposure without MIC change, driven by slow growth, low metabolism or altered membranes. Persistence is a stochastic, reversible dormant subpopulation that survives supra‐MIC doses and regenerates a susceptible population after antibiotic removal [95, 97]. EPS produces dispersal‐reversible tolerance; persisters enrich in interiors via starvation, hypoxia, TA systems and the stringent response; HGT‐permissive, mutation‐elevated conditions drive inherited resistance. Consequently, nifH or compositional shifts under As or antibiotic stress cannot be assigned to a single tier without genetic, single‐cell and population data [96].

Penetration restriction operates via multiple routes. Anionic EPS—eDNA and uronic‐acid polysaccharides—electrostatically sequester aminoglycosides, creating subinhibitory gradients [96, 98]. Matrix‐retained *β*‐lactamases hydrolyse *β*‐lactams before they reach deep cells. Fluoroquinolones diffuse freely but lose ROS‐mediated killing at low O_2_ [98, 99]. Metabolic dormancy provides a second layer: *β*‐lactams, fluoroquinolones, aminoglycosides, macrolides and rifampicin all require active wall synthesis, gyrase, ribosomes or RNA polymerase, so quiescent interior cells are intrinsically tolerant [97]. Critically, nitrogenase‐expressing low‐O_2_ interior cells occupy the same dormant, tolerant zone. Active efflux is a third route: *P. aeruginosa* RND systems MexAB‐OprM, MexCD‐OprJ, MexXY‐OprM and MexEF‐OprN are upregulated in biofilms and under sublethal exposure [100]; MexR is inhibited by pyocyanin, with NalD, PA3898 and PA2100 adding control [101], linking phenazine‐mediated electron transfer to elevated multidrug efflux.

Biofilms also evolve inherited resistance via elevated mutation supply, accelerated HGT and selective enrichment [98]. SOS—triggered by ssDNA‐driven RecA filaments allosterically promoting LexA autocleavage, resolved as a three‐RecA interface [102]—derepresses > 40 genes including Pol IV (DinB) and Pol V (UmuDC), raising mutation rates from ~10^−9^ to ~10^−4^ per base per division [40]. SOS is amplified by sublethal antibiotic gradients, As‐induced ROS lesions (8‐oxoguanine, AP sites) and eDNA‐rich RecA templates; it also activates Class 1 integron integrases, mobilising ARG cassettes [103]. The stringent response—(p)ppGpp by RelA/SpoT—suppresses rRNA, redirects transcription and promotes persisters via TA overlap [104]; nitrogen limitation simultaneously derepresses nifA and triggers (p)ppGpp, producing cells that fix N_2_, enter dormancy and elevate SOS together. Deleting up to 10 Type II TA operons cut *E. coli* persisters 100–200‐fold [105, 106]; HipAB drives biofilm tolerance; SOS‐induced TisAB/IstR‐1 depletes ATP, conferring fluoroquinolone tolerance and *S. aureus* biofilm persisters elevate TA expression after bactericidal exposure [107].

Clinical biofilm models translate only partially to environmental settings. Clinical biofilms are mono‐/oligospecies in nutrient‐rich fluids at 37°C under supra‐MIC dosing; environmental biofilms are multispecies under fluctuating subinhibitory exposures (nM–low *μ*M) that maximise SOS‐driven mutagenesis without killing [103]. The environmental resistome is far larger, with As and heavy‐metal resistance genes (MGRs) colocated with ARGs on shared MGEs, enabling metal‐selected ARG persistence absent clinically [108]. Most distinctively, N‐fixing environmental biofilms generate interiors—low O_2_, high c‐di‐GMP, elevated (p)ppGpp, eDNA‐rich, AHL‐saturated—simultaneously optimal for nitrogenase function, persister formation, SOS induction and HGT, justifying treatment of nitrogen fixation, As transformation and AR as one integrated system.

## 6. As‐Driven Coselection of AR

### 6.1. Co‐Occurrence of ARGs, MRGs, Cross‐Resistance, Coresistance and Coregulation Frameworks

As and heavy metals maintain resistance without antibiotic pressure by colocating MRGs and ARGs on shared MGEs (Figure 4). Metals do not degrade, exerting more persistent selection than antibiotic residues. In 50 urban soils, metal toxicity indices (Cd, As, Zn, Cu and Pb) predicted ARG number and abundance, with path analysis showing MGE proliferation—not chromosomal selection—mediated the effect [37]. Poultry‐farm soils showed As‐correlated ARG/MGE coenrichment and resistome–community costructuring [38]. In As‐contaminated paddy soils, qPCR confirmed As raised ARG and MGE abundance, manure raised ABG abundance and ABG–ARG co‐occurrence rose under As stress with multidrug ARGs preferentially associated; *Bacillus* and *Clostridium* were dominant co‐hosts [6]. Sulfamethoxazole coexposure (10–50 mg kg^−1^) raised ARGs, MGEs and HMRGs, MGEs being dominant mediator—confirming HGT [109, 110]. In Lake Macquarie sediments, two‐thirds of plasmids from metal‐impacted sites carried both MRGs and ARGs, with phenotype matching genotype.

**Figure 4:**
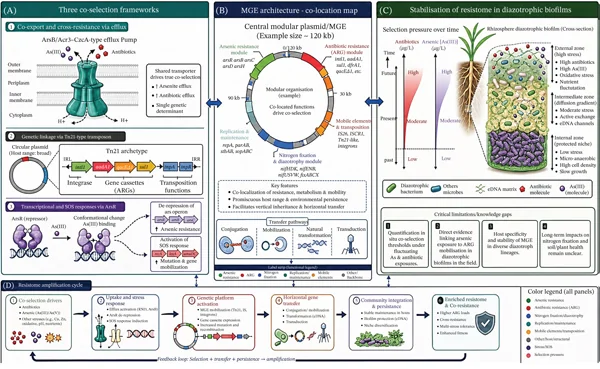
Arsenic‐driven coselection of antibiotic resistance in diazotrophic biofilms. (A) Three nonmutually exclusive coselection frameworks: cross‐resistance via ArsB‐mediated PMF depletion; coresistance through *arsRBC*–ARG colocation on shared Tn21‐type MGEs; and coregulation via the global ArsR stimulon and As(III)‐induced ROS → RecA → LexA → *intI1* SOS cascade. (B) The conceptual diagram assembles onto one hypothetical replicon a set of modules that have been documented separately: ars–ARG colocation on broad‐host‐range IncP/IncQ/IncW plasmids, including arsRBC on Tn402 within IncP‐1*α* alongside aadB and bla; Tn21‐type transposons and Class 1 integron cassettes; and nifHDK/fixABCX clusters with metal‐tolerance determinants on rhizobial pSym replicons. (C) Arsenic as a persistent, nondegrading coselector stabilising the resistome in diazotrophic rhizosphere biofilms beyond antibiotic pressure—with four unresolved evidentiary limitations: correlation–causation gap, gene‐expression–phenotype decoupling, metal co‐variance and clinical risk translocation. (D) Resistome amplification sample.

Three nonmutually exclusive frameworks explain As‐driven resistance selection (Figure 4A) [111]. Cross‐resistance arises when one gene product handles both classes without linkage. The best characterised examples of metal–antibiotic cross‐resistance involve cation efflux: CzcCBA in *Cupriavidus metallidurans* confers cobalt, zinc and cadmium resistance and MexAB‐OprM in *P. aeruginosa* is a broad‐substrate multidrug pump. These systems establish the principle that a single efflux apparatus can generate cross‐resistance, but we identified no demonstration that either extrudes As(III) or As(V), or that either mediates As‐specific antibiotic coselection. As‐specific efflux is instead mediated by the dedicated ArsB and Acr3 permeases, which are structurally distinct from RND pumps, belong to separate transporter families and are not multidrug transporters. Where As(III) exposure has been associated with reduced *β*‐lactam susceptibility in *E. coli*, the proposed mechanism is indirect, acting through perturbation of the proton‐motive force rather than through shared substrate handling and requires independent confirmation. Non‐specific stress responses, in particular As‐induced ROS generation and the resulting RecA/LexA‐dependent SOS response, are similarly generic to oxidative and genotoxic stress. Coresistance arises when *ars* and ARGs colocate on the same plasmid, transposon or integron, so As selection drags ARGs along. Class 1 integrons frequently carry As cassettes alongside ARGs; Tn21 (*merA*, *merB*, *sul1* and *qacE*) is archetypal, and *arsRBC*‐bearing integrons occur in river sediment and manure soils [36]. Plasmid‐resolved metagenomics confirms *arsRBC* and ARG cassettes share replicons far above background [112]. Coregulation arises when shared promoters or stress responses induce both sets together. The ArsR stimulon in *A. tumefaciens* 5A includes multidrug, heavy‐metal and efflux‐pump genes alongside *ars* genes [33, 113]; As‐induced ROS activates RecA/LexA‐driven SOS, derepressing *intI1* and accelerating ARG cassette rearrangement [103, 114]. In drinking‐water biofilms, resistant bacteria were coselected within 6 h at 0.2–1 mg/L As(III) and persisted ≥ 4 days after As removal [39]. Figure 4B depicts a representative ~120 kb modular plasmid/MGE in which As resistance genes (*arsR, arsB, arsC, arsD* and *arsH*), AR determinants (*intI1*, *aadA1, sul1, dfrA1* and *qacE* *Δ*1), nitrogen fixation genes (*nifHDK, nifENB, nifUSVW* and *fixABCX*) and mobility/transposition elements (IS26, ISCR1 and Tn21‐like integrons) are physically colocated on a single replicon alongside core replication and maintenance machinery (r*epA, parAB, stbAB* and *sopABC*). This genomic architecture indicates that As resistance, AR and diazotrophy are not independently distributed traits but are genetically linked on a common, broad‐host‐range mobile platform capable of dissemination via conjugation, mobilisation, natural transformation and transduction. Consequently, selection imposed by any single stressor—whether As, antibiotics or nitrogen limitation—can coselect for the entire linked cassette, providing a plausible mechanistic basis for the co‐occurrence of these traits observed in diazotrophic biofilm communities, though direct in situ evidence confirming this comobilisation under field‐relevant, fluctuating stress conditions remains a key knowledge gap.

### 6.2. How As Stress May Stabilise Resistomes in Diazotrophic Biofilms and Limits of Existing Evidences

Nitrogen‐fixing rhizosphere biofilms simultaneously experience (i) As loading; (ii) agricultural antibiotic input; (iii) high cell densities, eDNA and plasmid transfer; and (iv) diazotrophy‐favouring conditions (Figure 4C,D)—making As‐contaminated hyperaccumulator rhizospheres potentially the most intensively coselected ARG reservoirs outside wastewater systems. In paddy soils, ABG–ARG coenrichment peaked under combined As plus manure, with diazotroph‐related *Bacillus* and *Clostridium* as dominant co‐hosts [6]. In hyperaccumulator rhizospheres, *Rhizobiales* and *Burkholderiales* dominate As‐enriched diazotrophs; both carry modular plasmids combining symbiotic, nitrogen‐fixation and resistance cassettes, enabling single‐replicon coselection of diazotrophy, As resistance and AR. Crucially, As provides persistent, nondegrading pressure preventing ARG loss after antibiotic cessation: Residues decompose in weeks to months, but iron‐oxyhydroxide‐bound As is released under biofilm‐driven reducing conditions, continuously reselecting ARG‐bearing members [37]. Even with antibiotic use stopped, ARGs in As‐contaminated biofilms would stay elevated via plasmid‐ and integron‐mediated coselection [39].

• Correlation–causation gap. As–ARG co‐occurrence is consistent with coselection but does not prove it [111]. Alternatives include independent selection by pH, organic matter or land use; preservation of high‐GC organisms; and biomass artefacts. Functional validation—MIC shifts or *ars* + ARG plasmid enrichment versus matched controls—covers only a small fraction of studies.
• Gene detection versus expression vs phenotype. qPCR and metagenomics detect DNA, not mRNA or phenotypes [6]. Paddy metatranscriptomics showed *ars* transcription rose two‐ to fivefold above 410 mg kg^−1^ As, yet DNA abundance was unchanged below that threshold [115]—implying many qPCR correlations reflect historical accumulation, and co‐expressed *ars*/ARG mRNA is needed for mechanistic claims.
• Selectivity versus co‐occurring metals. As rarely occurs alone; Cd, Zn, Cu, Pb, Hg and Sb co‐vary, and Cu/Zn are stronger ARG coselectors than As in manure soils [38]. As‐specific claims require separating it from co‐varying metals and demonstrating As‐specific mechanisms (*ars*–ARG colocation and ArsR coregulation) rather than generic stress.
• Ecological risk translation. Not all environmental ARGs threaten clinical therapy: Many share only distant homology with clinical genes, target non‐medical antibiotics or are nontransferable [108, 116]. Risk depends on transferability to human pathogens and exposure pathways (e.g., rice or leafy vegetables), neither characterised for most diazotrophic biofilm ARG reservoirs.

### 6.3. The Nitrogen Fixation–AR Duo: Direct Evidence, Opposing Signs and the Remaining Gap

The two limbs considered so far are asymmetrically supported: As–resistance coselection rests on genetic colocation and As–diazotrophy interaction on transcript‐level measurements. The third limb, nitrogen fixation and AR, is usually inferred from As as a shared selector or spatial coincidence in low‐oxygen biofilm interiors—neither of which demonstrates a pairwise relationship, and the shared‐selector argument is circular if As is also under study. Genomic linkage in shared hosts: Diazotrophy and AR are frequently coencoded, so selection on either acts on both by linkage alone. All 14 legume nodule–associated *Klebsiella oxytoca* isolates carried complete nif operons alongside averaging 29 resistance determinants, 71% multidrug‐resistant [117]. Complete nif clusters segregate with phylogroups circulating clinically [118], and a human‐colonising *Klebsiella* isolate carried nif genes alongside novel *β*‐lactamase alleles [119]. Resistance determinants are widespread among plant‐growth‐promoting inoculants [120], and ampicillin resistance was cloned from *A. brasilense* Sp7 [121]—linkage, not interaction, but testable via long‐read resistome quantification.

Resistance determinants selected by the symbiotic niche: ARG abundance decreased from nodule to rhizosphere to bulk soil, with efflux genes dominant and rhizobia principal hosts [72]. In *S. meliloti*, the Fsr transporter confers antimicrobial resistance and is required for effective symbiosis—loss lowered fosfomycin MIC drastically and reduced nodulation and plant mass, without altering nitrogenase activity [122]. EmrR is required for *Medicago sativa* nodule colonisation [123], and BdeAB is host‐specific in *Bradyrhizobium japonicum* [124]—efflux capacity maintained by plant–microbe interface chemistry independent of anthropogenic antibiotics or As. Shared regulators couple nitrogen status to antibiotic outcome: RpoN, obligatory for nif transcription, alters quinolone/carbapenem susceptibility and tobramycin survival in *P. aeruginosa* [125–127]. NtrBC deletion lowered ciprofloxacin MIC 16‐fold [128], and glnA inactivation increased quinolone susceptibility via OmpF [129]. Nitrogen‐starved *E. coli* generated 20‐fold more NtrC‐dependent persisters [130]; (p)ppGpp promotes biofilm antibiotic tolerance [131]. Anr, required for nifH/nifC expression and biofilm formation [28], is also required for biofilm ciprofloxacin tolerance in *P. aeruginosa* [132]—predicting coordinated shifts across rpoN, ntrC, relA/spoT and anr mutants.

Energetic coupling (prediction): nitrogenase′s high ATP demand and PMF‐dependent aminoglycoside uptake [133, 134] suggest diazotrophy in microoxic biofilms could lower aminoglycoside uptake without resistance genes—untested in diazotrophs, classified accordingly. Single proteins serving both functions: In *Anabaena* sp. PCC 7120, HgdD enables both heterocyst glycolipid deposition and antibiotic/xenobiotic export; mutants showed ~100‐fold lower export and 25‐fold higher accumulation [135], paralleling Fsr—establishing mechanistic inseparability without As or spatial co‐occurrence. The relationship can also be antagonistic, as phage resistance trades off against heterocyst function, shaped by nitrogen availability [44, 136]. The reciprocal direction is sign‐variable: Sulfadiazine reduced nifH abundance in clover rhizospheres [137], tetracycline reduced soybean fixation [138], yet oxytetracycline/sulfamethazine increased fixation 12.4%–20.8% [139], and veterinary antibiotics restructured rhizobial populations without direct toxicity [140]—interaction through community composition, sign depending on compound, dose and host. Fixed nitrogen feeds back on gene mobility: Ammonium inhibited plasmid conjugation in *Rhizobium meliloti* by two orders of magnitude [141], suggesting local suppression of conjugation in diazotrophically active biofilms. Field nitrogen inputs reshape resistomes variably [142], suggesting ammonium‐mediated regulation alongside selection—a discriminating test. What is missing: No study has commeasured fixation activity and ARG abundance in the same environmental system, except strain‐level fsr work [122]. Until then, the nif–AR limb is closed by genomic linkage, niche‐selected efflux, shared regulation and ammonium‐modulated mobility, with As as one contributing route, not the bridge the triad depends upon.

## 7. HGT in As‐Exposed Diazotrophic Biofilms

### 7.1. Conjugation, Transformation, Transduction and eDNA Retention in Biofilm Settings

HGT redistributes AR, metal resistance and diazotrophy across distant lineages, outpacing chromosomal mutation [143, 144] (Figure 5). Biofilms amplify HGT (Figure 5A): EPS proximity supports conjugation/OMV transfer; eDNA matrix sustains competence; subinhibitory As/antibiotic gradients trigger SOS and integrase expression; QS upregulates *tra* [145]. Conjugation dominates, with biofilm transfer 700‐fold above liquid culture [41]; T4SS pili prolong donor–recipient contact under EPS confinement [146]. Li et al. [147] tracked RP4 (IncP‐1*α*, aminoglycoside/*β*‐lactam) in *P. putida*–*E. coli* biofilms: Intraspecies initiation triggered a retransfer‐dominated chain‐reaction cascade, with > 46% of activated‐sludge genera permissive. Transformation rises in early biofilms via matrix‐eDNA‐induced competence (*Streptococcus*, *Bacillus*, *Haemophilus*; inducibly *P. aeruginosa com* regulon), mobilising nonconjugative plasmids and chromosomal ARGs [143, 146]. eDNA from lysed diazotrophs at the oxic–anoxic interface—carrying *nif*, *ars* and ARG cassettes—concentrates where competence peaks, enabling joint acquisition of diazotrophy and resistance. Transduction acts via generalised (~50 kb, ~10^−8^/gene/burst) and specialised routes; EPS slows phage diffusion, protecting interior cells while outer cells amplify transducing particles. OMVs (30–250 nm) contact‐independently deliver DNA, resisting exonucleases. Global plasmid–phage ARG dissemination from WWTPs (MLS, *β*‐lactam) confirms OMV/phage complement conjugation [148].

**Figure 5:**
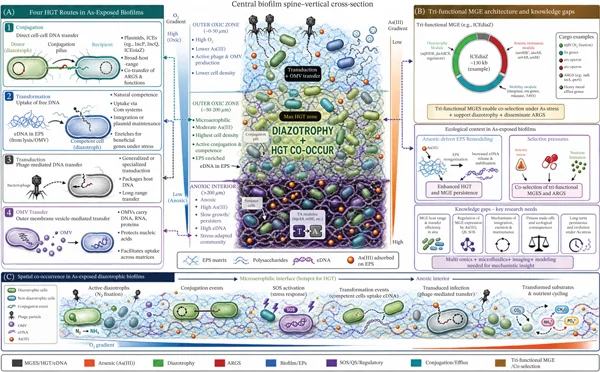
Horizontal gene transfer in arsenic‐exposed diazotrophic biofilms. (A) Four HGT routes and their proposed route‐dependent spatial optima across the biofilm oxygen gradient. The spatial assignments in this panel are hypothetical. Conjugation (~700× planktonic rate; T4SS pili stabilised by EPS confinement; QS → *tra* upregulation); eDNA‐mediated transformation peaking at the oxic–anoxic interface where competence induction and lysis‐driven eDNA release overlap; phage transduction attenuated by EPS diffusion barriers and therefore relatively more important nearer the surface; and OMV‐mediated contact‐independent ARG delivery, the least spatially constrained route. (B) Hypothetical trifunctional ICE/plasmid architecture diagram indicates where ars operons (Tn21/Tn402), ARG cassettes (intI1–sul1–aadA–blaTEM) and nifHDK/fixABC would have to reside for a single element to be coselected by arsenic, antibiotic and diazotrophic pressures. Candidate host lineages in Rhizobiales/Burkholderiales/*Paenibacillus* are those in which ars–ARG or ars–nif colocation has separately been reported; co‐occurrence of all three on one replicon in these hosts has not been confirmed by long‐read assembly; arsenic dose‐dependently remodels EPS towards eDNA/uronate enrichment, modulating transformation independently of SOS. (C) Co‐occurrence of nitrogenase activity, conjugation, SOS → *intI1*, QS and (p)ppGpp in one anoxic interior zone—three unresolved gaps (spatial, donor–recipient and long‐read trifunction confirmation) highlighted.

EPS provides scaffold, chemistry and regulatory cues for HGT [22]. Matrix eDNA accumulates from attachment secretion, SOS‐induced phage lysis and anaerobic turnover—biased towards replication‐terminus regions, prophage flanks and diazotroph genomes. Sun et al. [43] showed in vivo EPS depletion in *P. aeruginosa* cut conjugation despite higher membrane permeability/ROS—ATP fell, *traE*/*traI*/*traJ* transcription declined and donor–recipient pairings dispersed—proving EPS′s physical‐energetic, not chemical‐exclusion, role. Under As, EPS is remodelled: Tournay et al. [42] showed 50 *μ*M As(V) in *Pseudomonas* PD9R and *Rahnella laticis* PD12R raised uronic acid and eDNA relative to polysaccharide while maintaining biomass; 500 *μ*M suppressed both. eDNA‐enriched matrices would elevate transformation while sustaining conjugation—a SOS‐independent pathway by which As raises HGT, untested versus direct ArsR/SOS effects. QS adds a second layer: AHL systems (*lasI/rhlI–lasR/rhlR*) upregulate *pel*, *psl*, eDNA release and *tra* [11, 83, 84]. QSIs vanillin and Acylase I cut conjugation to 0.32‐/0.13‐fold in *E. coli*–*P. aeruginosa*. In diazotrophic biofilms, elevated AHL coactivates N_2_ fixation, EPS production and ARG transfer.

### 7.2. Potential Colocation Among *ars* Genes, ARGs and Diazotrophy‐Associated Functions on Mobile Elements and the Unresolved Problem of Donor–Recipient Identity

Colocation of *ars*, ARGs and *nif* on shared plasmids; ICEs; or genomic islands would create three‐way units coselected by As, antibiotic and diazotrophic pressures (Figure 5B,C)—stabilising resistomes without one selector. At the *ars*–ARG level, *arsRBC*/*arsRBCMH* colocate with ARGs on broad‐host IncP/IncQ/IncW plasmids [35, 36]; IncP‐1*α* carries *arsRBC* on Tn402 alongside *aadB* and *bla*. At the *ars*–*nif* level, *S. fredii*, *B. japonicum* and *M. loti* pSym plasmids encode *nifHDK*, *fixABCX* and *nodABC* with metal‐tolerance genes; AqpS is adjacent to *S. meliloti* symbiotic clusters [81]. In paddy soils, *Bacillus*/*Clostridium*—including *Paenibacillus* carrying *nifH* with *arsBC*—are dominant ABG/ARG co‐hosts [6]. Assembly‐based signals consistent with trifunctional cargo have been reported from functional metagenomics of As‐contaminated rhizospheres, in which ars, ARG and plant‐growth‐promoting (including nif) determinants coassemble on plasmid contigs [35], and As enriches 12 of 17 ARG types in the same systems. These are contig‐level coassemblies, however, and we are not aware of any closed, circularised replicon in which ars, ARG and nif determinants have been confirmed together by long‐read sequencing with independent breakpoint verification, nor of any isolate in which all three functional categories have been shown to transfer as a single unit. The landscape comprises Class 1 integrons, composite transposons flanking *arsRBC*/*arsM* and ICEs [149, 150]; ICEs are the most plausible tricargo vehicles given large capacity and SOS‐elevated biofilm transfer.

The central gap is spatially resolved, donor–recipient‐trackable HGT in realistic N_2_‐fixing biofilms—needed to confirm whether overlap of diazotrophic, high‐HGT and As stress zones generates community‐scale *ars*–ARG–*nif* transfer. Current methods are limited: Bulk assays confirm 10–1000‐fold biofilm‐over‐planktonic transfer but are spatially blind; microfluidic imaging [147] gives spatial data only in two‐species models; FACS estimates HGT versus vertical contribution in WWTPs without spatial mapping [151]. Bacterial‐MERFISH [152]—multiplexed FISH with 1000‐fold expansion—profiles ~80% of the *E. coli* transcriptome at single‐cell, single‐molecule resolution and demonstrated for carbon starvation and *B. thetaiotaomicron* gut niches, with biofilm persister/resistance mapping as priorities. Applied to As‐exposed diazotrophic biofilms with tagged *ars*–ARG plasmids, it would colocate *nifH*‐, *arsR*‐ and *tra*‐expressing cells. Par‐seqFISH ([153], *Science*) on *P. aeruginosa* biofilms (600,000 transcriptomes) showed micrometre‐distinct states coexist. Correlative NanoSIMS with ^15^N_2_, As‐isotope and ^13^C labelling [154] maps fixation and As transformation subcellularly. Within the literature searched, we did not identify a study in which bacterial‐MERFISH or par‐seqFISH had been combined with NanoSIMS in the same biofilm; this remains, to our knowledge, a feasible programme yielding the first integrated map of diazotrophy, As transformation, ARG expression and HGT.

## 8. Future Research Agenda

### 8.1. Minimum Mechanistic Dataset (MMD) and Cell‐Resolved Causal Inference for Biofilm‐Mediated Nitrogen Fixation Under As Stress

The field needs an explicit MMD for studies on biofilm‐mediated N_2_ fixation under As. Existing work mostly reports correlations among As, *nifH* gene/transcript abundance, diazotrophic community composition and soil N indicators—insufficient to distinguish mechanistic alternatives with divergent management implications. The MMD has six tiers: (i) N_2_ fixation confirmed by ^15^N_2_ incorporation (IRMS) or acetylene reduction across As gradients—*nifH* abundance/transcripts alone are insufficient as 323/1352 *nifH* ^+^ genomes lack complete *nifHDK*, paralogs are widespread and single‐cell heterogeneity decouples bulk transcripts from rates [43]; (ii) Fe‐protein activity and DraT‐mediated ADP‐ribosylation at NifH‐Arg101, distinguishing post‐translational inactivation (hours) from transcriptional downregulation (days) or metalloprotein destruction; (iii) Fe–S cluster integrity by EPR on anaerobic lysates, separating [4Fe–4S] disruption from NifL‐redox effects on NifA; (iv) *nif*–*ars* coprofiling by qRT‐PCR/RNA‐seq, ideally spatially via bacterial‐MERFISH [152], testing coregulation, trade‐off or independence; (v) As speciation/redox microenvironment by O_2_/redox microsensors and synchrotron‐XRF As‐mapping on the same sections; and (vi) reversibility under As removal, separating tolerance, regulatory memory and genetic resistance—outcomes demanding different interventions.

Bulk metagenomic, metatranscriptomic and qPCR markers average over cells of differing identity, position and state. Bacterial‐MERFISH [152] profiles thousands of operons per cell at micrometre resolution, mapping *nifH*, *arsR*, *tra*, SOS (*recA*, *dinB* and *umuC*) and TA (*hipA* and *tisB*) co‐expression to test whether diazotrophic, As stress and HGT states converge in the same cells. Par‐seqFISH [153] profiled 600,000 *P. aeruginosa* cells, resolving metabolic states 5 *μ*m apart. Pairing with SIP‐NanoSIMS (^15^N_2_, ^75^As) yields an integrated functional–transcriptional atlas of diazotrophic, As‐accumulating and HGT‐active cells [154], echoing legume‐nodule spatial atlases [155].

### 8.2. Comparative Models, EPS‐Mediated HGT Mechanisms and the Resistance–Diazotrophy Trade‐Off

A three‐tier model framework is needed. Monocultures (*P. stutzeri* A1501, *E. coli* and *A. tumefaciens* 5A) dissect NifA/NifL/RpoN, ArsR stimulon and c‐di‐GMP–*nif* regulation [28, 33] but miss community emergent properties. Synthetic communities (2–10 species) probe crossfeeding, coresistance and HGT topology; a minimal four‐member design—As‐resistant *nifH* ^+^ backbone (*P. stutzeri*/*Paraburkholderia*), *arsM* ^+^ SAM partner, ARG‐carrying conjugation‐active strain and carbon‐providing nondiazotroph—tests SAM‐coupling and conjugative‐transfer hypotheses. Environmental microcosms (rhizosphere, paddy and sediment) need As gradients (not single doses), ^15^N_2_ rates (not *nifH* alone), paired ARG/*ars* abundance and weeks to months durations. Comparative analysis across all three levels of one regulatory question has not been done. Three pathways could explain As‐elevated biofilm HGT: (i) SOS—As → ROS → DNA damage → RecA → integrase induction, testable in *recA*‐null backgrounds; (ii) EPS remodelling—As alters *psl*/*pel*/eDNA‐release expression towards eDNA‐ and uronic‐acid‐richer matrices [42], testable in *pslΔ*/*algDΔ*/*pelBΔ* mutants; (iii) community turnover—As‐driven enrichment of high‐HGT taxa (*Burkholderiales*), resolvable by species‐tagged MGE tracking with FACS/single‐cell sequencing. Sun et al. [43] showed EPS depletion reduces conjugation despite higher ROS/permeability—an SOS‐independent EPS effect grounding pathway (ii).

The resistance–diazotrophy trade‐off is context‐dependent. At single‐cell level, SAM/energy costs of As detoxification and ARG expression (efflux pumps, *β*‐lactamases and TA antitoxins) compete with ATP‐intensive nitrogenase: Phage resistance in cyanobacteria impairs heterocyst function [44]; comparative genomics across 15,000+ genomes shows N_2_‐fixation gene content is shaped by ecological constraints incompatible with sustained resistance selection [45]. At community level, labour division—*arsM* ^+^ nondiazotrophs absorbing the SAM burden, diazotrophs in EPS‐protected interiors, ARG‐carrying peripheral cells buffering antibiotics—can resolve it, predicting positive correlation between complexity and joint nitrogenase/ARG load (testable with two, four, eight and 16‐member consortia). Engineered As‐resistant, N_2_‐fixing consortia for paddy/hyperaccumulator rhizospheres should combine *arsM* ^+^ strains, ARG‐bearing matrix formers and efficient diazotrophs with minimal *ars* load.

## 9. Conclusion

Biofilms constitute the essential molecular reactor in which nitrogen fixation, As transformation and AR may operate as an interacting system rather than as three independent processes. EPS‐generated oxygen gradients, eDNA‐rich matrix chemistry, c‐di‐GMP–RpoN–Gac/Rsm coregulation and stress‐induced SOS/integrase activity converge on the same anoxic interior cells, producing the only ecological context where ATP‐intensive diazotrophy, ArsM‐driven methylation, persister survival and elevated HGT co‐occur. As acts as a nondegrading coselector that stabilises ARGs on plasmids, integrons and ICEs long after antibiotic input ceases, whereas diazotrophy‐derived ammonium fuels SAM provisioning for As detoxification—creating a direct consequence for soil nitrogen fertility, food‐chain As exposure and environmental dissemination of clinically relevant resistance. If closed, this would constitute a self‐reinforcing triad, but closure has not been demonstrated experimentally. Mechanistic causality remains underresolved: *nifH* presence does not imply N_2_ fixation, qPCR does not capture expression heterogeneity, and *ars*–ARG co‐occurrence does not prove coselection. Resolving these gaps demands the proposed MMD (^15^N_2_ rates, DraT modification, Fe–S integrity, paired *nif*–*ars* transcriptomics, redox microsensors and reversibility) coupled with bacterial‐MERFISH, par‐seqFISH and SIP‐NanoSIMS in synthetic communities and As‐contaminated rhizospheres. Such integrative resolution will translate biofilm crosstalk into actionable interventions for sustainable agriculture, As‐contaminated rhizosphere remediation and one‐health resistance mitigation while informing rational design of engineered N_2_‐fixing consortia tolerant to metalloid cocontamination.

## Author Contributions

A.M.: conceptualisation, write‐up, figure preparation and revision. D.B.: write‐up and revision. I.K‐L. and M.B.: revision. D.B. and A.M. have contributed equally to this work.

## Funding

No funding was received for this manuscript.

## Conflicts of Interest

The authors declare no conflicts of interest.

## Data Availability

Data sharing not applicable to this article as no datasets were generated or analysed during the current study.
